# Heterogeneous drug penetrance of veliparib and carboplatin measured in triple negative breast tumors

**DOI:** 10.1186/s13058-017-0896-4

**Published:** 2017-09-11

**Authors:** Imke H. Bartelink, Brendan Prideaux, Gregor Krings, Lisa Wilmes, Pei Rong Evelyn Lee, Pan Bo, Byron Hann, Jean-Philippe Coppé, Diane Heditsian, Lamorna Swigart-Brown, Ella F. Jones, Sergey Magnitsky, Ron J Keizer, Niels de Vries, Hilde Rosing, Nela Pawlowska, Scott Thomas, Mallika Dhawan, Rahul Aggarwal, Pamela N. Munster, Laura J. Esserman, Weiming Ruan, Alan H. B. Wu, Douglas Yee, Véronique Dartois, Radojka M. Savic, Denise M. Wolf, Laura van ’t Veer

**Affiliations:** 1 Department of Medicine, University of California San Francisco, 2340 Sutter Street, San Francisco, CA 9411 USA; 2 Rutgers New Jersey Medical School, Public Health Research Institute, Rutgers, The State University of New Jersey, 225 Warren Ave, Newark, NJ USA; 3 Department of Pathology, University of California, San Francisco, CA USA; 4 Department of Radiology and Biomedical Imaging, University of California, San Francisco, CA USA; 5 Department of Laboratory Medicine, UCSF Helen Diller Family Comprehensive Cancer Center, San Francisco, CA USA; 6 UCSF Helen Diller Family Comprehensive Cancer Center, San Francisco, CA USA; 7 Department of Bioengineering & Therapeutic Sciences, University of California San Francisco, San Francisco, USA; 8 Patient advocate University of California, San Francisco Breast Science Advocacy Core, San Francisco, CA USA; 9 Department of Laboratory Medicine, University of California San Francisco, San Francisco, CA USA; 10 Division of Hematology Oncology, University of Minnesota, Minneapolis, MN USA; 11 Department of Clinical Pharmacy, Department of Pharmacy & Pharmacology, The Netherlands Cancer Institute, NKI-AVL, Amsterdam, The Netherlands

**Keywords:** Drug penetration, Spatial heterogeneity, Pharmacokinetics, Matrix-assisted laser desorption/ionization mass spectrometric imaging, Poly(ADP-ribose) polymerase inhibitors, Carboplatin, Inductively coupled plasma–mass spectrometry

## Abstract

**Background:**

Poly(ADP-ribose) polymerase inhibitors (PARPi), coupled to a DNA damaging agent is a promising approach to treating triple negative breast cancer (TNBC). However, not all patients respond; we hypothesize that non-response in some patients may be due to insufficient drug penetration. As a first step to testing this hypothesis, we quantified and visualized veliparib and carboplatin penetration in mouse xenograft TNBCs and patient blood samples.

**Methods:**

MDA-MB-231, HCC70 or MDA-MB-436 human TNBC cells were implanted in 41 beige SCID mice. Low dose (20 mg/kg) or high dose (60 mg/kg) veliparib was given three times daily for three days, with carboplatin (60 mg/kg) administered twice. In addition, blood samples were analyzed from 19 patients from a phase 1 study of carboplatin + PARPi talazoparib. Veliparib and carboplatin was quantified using liquid chromatography–mass spectrometry (LC-MS). Veliparib tissue penetration was visualized using matrix-assisted laser desorption/ionization mass spectrometric imaging (MALDI-MSI) and platinum adducts (covalent nuclear DNA-binding) were quantified using inductively coupled plasma–mass spectrometry (ICP-MS). Pharmacokinetic modeling and Pearson’s correlation were used to explore associations between concentrations in plasma, tumor cells and peripheral blood mononuclear cells (PBMCs).

**Results:**

Veliparib penetration in xenograft tumors was highly heterogeneous between and within tumors. Only 35% (CI 95% 26–44%), 74% (40–97%) and 46% (9–37%) of veliparib observed in plasma penetrated into MDA-MB-231, HCC70 and MDA-MB-436 cell-based xenografts, respectively. Within tumors, penetration heterogeneity was larger with the 60 mg/kg compared to the 20 mg/kg dose (RSD 155% versus 255%, *P* = 0.001). These tumor concentrations were predicted similar to clinical dosing levels, but predicted tumor concentrations were below half maximal concentration values as threshold of response. Xenograft veliparib concentrations correlated positively with platinum adduct formation (*R* ^2^ = 0.657), but no PARPi–platinum interaction was observed in patients’ PBMCs. Platinum adduct formation was significantly higher in five gBRCA carriers (ratio of platinum in DNA in PBMCs/plasma 0.64% (IQR 0.60–1.16%) compared to nine non-carriers (ratio 0.29% (IQR 0.21–0.66%, *P <* 0.0001).

**Conclusions:**

PARPi/platinum tumor penetration can be measured by MALDI-MSI and ICP-MS in PBMCs and fresh frozen, OCT embedded core needle biopsies. Large variability in platinum adduct formation and spatial heterogeneity in veliparib distribution may lead to insufficient drug exposure in select cell populations.

**Electronic supplementary material:**

The online version of this article (doi:10.1186/s13058-017-0896-4) contains supplementary material, which is available to authorized users.

## Background

Triple-negative breast cancer (TNBC) is an aggressive subtype of breast cancer, disproportionately affecting young African American and African women, with limited therapeutic options. TNBC frequently display homologous recombination deficiency and high genomic instability [1]. Therefore, veliparib, a poly (ADP-ribose) polymerase inhibitor (PARPi), is promising for the treatment of TNBC. Preclinical and early clinical studies suggest that combining PARPi with a DNA-damaging agent such as carboplatin may be more efficacious, compared to single-agent PARPi treatment, especially in patients without germline BRCA1/2 (gBRCA) mutations [2, 3]. Although Veliparib combined with carboplatin had significant efficacy in patients with TNBC receiving neoadjuvant treatment in the I-SPY 2 trial, approximately 42% of triple negative (TN) patients did not have pathologic complete response (pCR) to veliparib-based treatment [4].

Drug exposure from small molecules such as veliparib or carboplatin is often assumed to be relatively homogenous across diseased tissues. In current dose escalation study designs, treatment dosage or plasma exposures are directly correlated with outcomes (toxicity and efficacy) [5]. However, studies show that the distribution of small molecules in tumors is highly variable and may not correlate with dose or plasma concentrations [6, 7]. TNBC generally has aggressive biology with a high proliferation rate, relatively large tumor size and low microvessel density in the center of the tumor and the necrotic zones [8]. Therefore, the microenvironment of TNBC may contribute to the variability in the uptake and distribution of veliparib and carboplatin in tumors, resulting in inadequate response and ultimately drug resistance [9]. A preclinical study of olaparib, another PARPi, showed that abnormalities in the vasculature may hinder the penetration of PARPi [10]. In addition, carboplatin-adduct formation (the covalent binding of carboplatin to nuclear DNA) in tumors is highly variable between patients and may be more predictive of treatment response than carboplatin exposures in plasma [11, 12].

Liquid chromatography–mass spectrometry (LC-MS) is routinely used for quantifying drug concentrations in plasma and tissues. Matrix-assisted laser desorption/ionization mass spectrometric imaging (MALDI-MSI) can map the spatial distribution of drugs in the tissues, and has recently been used to assess detailed drug penetration in biological tissues [13–17]. Inductively coupled plasma mass spectrometry (ICP-MS) is a sensitive technique for the determination of metals and is frequently used to study platinum levels in various biological matrices such as ti*s*sues and peripheral blood mononuclear cells (PBMCs) from patients [12, 18, 19]. Dynamic contrast-enhanced magnetic resonance imaging (DCE-MRI) provides structural and functional information on tumor microvasculature [20–22]. It can be used to monitor perfusion/permeability of tumor vasculature/tissue and identify tissue areas to which drugs would be actively delivered.

Here we hypothesize that insufficient or heterogeneous veliparib penetration and platinum adduct formation in solid tumors may lead to inadequate response to PARPi/carboplatin in some patients with TNBC. As a first step toward testing this hypothesis, we performed a feasibility study using ICP-MS, LC-MS and MALDI-MSI to quantify and visualize the penetration of veliparib and carboplatin in TNBC mouse xenografts derived from three different TNBC cell lines and in PBMCs from patients. In this study we investigated the dependency of drug penetration on dosage and tumor characteristics in these TNBC mouse models and investigated potential drug–drug interactions between PARPi and carboplatin. We further visualized penetration of a contrast agent as a surrogate for diffusible hydrophilic compounds using a DCE-MRI pilot. If our hypothesis is correct, adjusting the dose to an individual patient’s tumor for increased penetration may lead to improved response and better patient outcomes.

## Methods

### Animal pharmacokinetic (PK) studies

In vivo experiments were performed with the approval of our Institutional Animal Care and Use Committee (IACUC). Three TNBC or claudin-low cell lines were chosen to reflect diverse tumor characteristics and sensitivities to single-agent PARPi therapies: MDA-MB-436 is a claudin-low mesenchymal-like, basal-B subtype BRCA1-mutated cell line, with a median PARP1 baseline expression (0.26) [23] showing sensitivity to olaparib [24] and veliparib [23, 25] in vitro. MDA-MB-231 is a mesenchymal-like, basal-B subtype TNBC, with low PARP1 baseline expression (−0.059) [23] with sensitivity to veliparib in vitro [23, 25] and in vivo [26, 27]. HCC70 is a basal-like, basal-A subtype TNBC, with high PARP1 baseline expression (0.544) [23]. These cells are insensitive to veliparib in vitro [23], but are sensitive to olaparib [24].

**Fig. 1:**
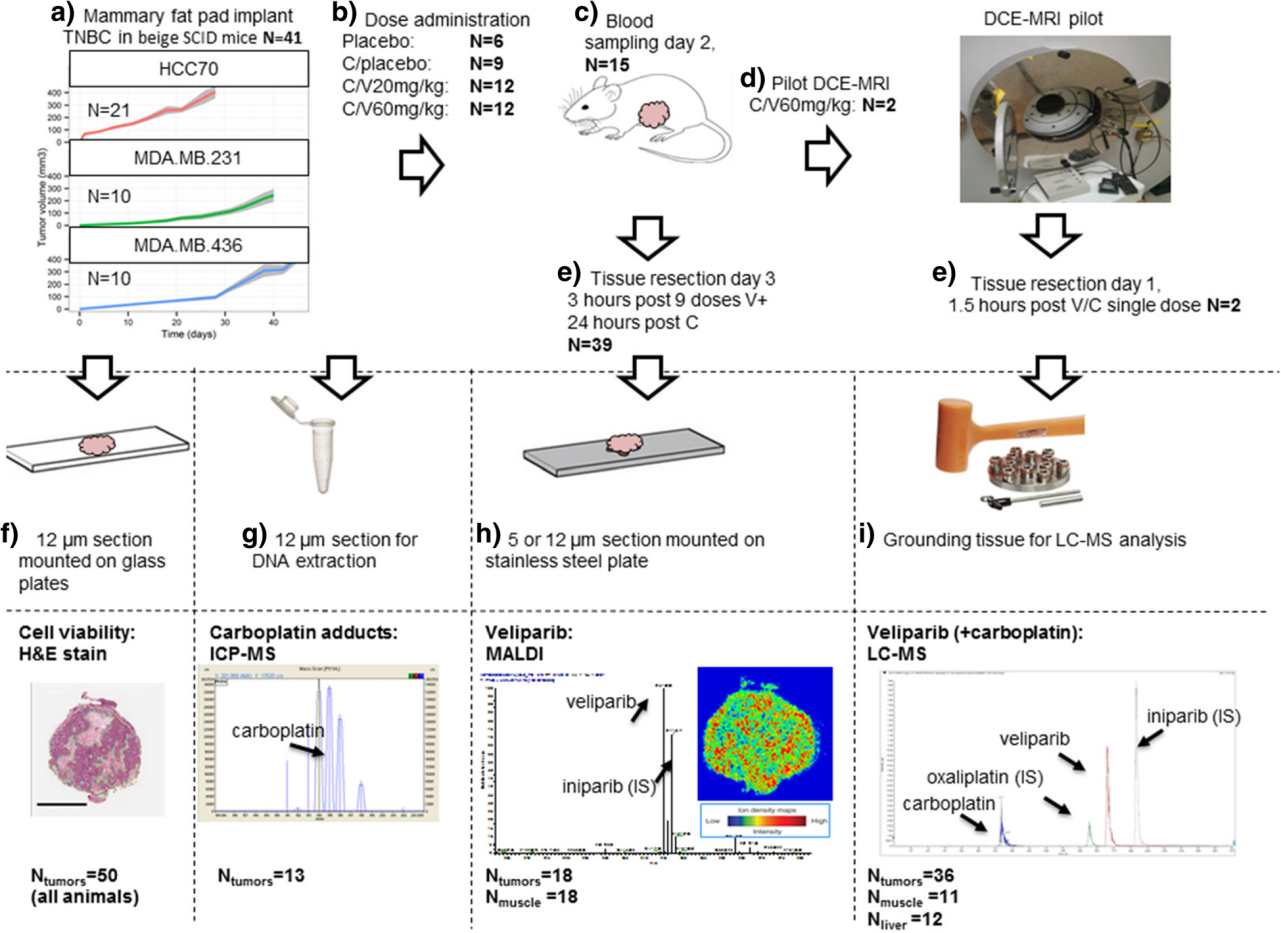
Study design. **a** Forty-one beige DF mice with severe combined immunodeficiency (SCID) were implanted with 10^6^ MDA-MB-231 (N = 10), HCC70 (N = 21) or MDA-MB-436 (N = 10) triple negative breast cancer (TNBC) cells bilaterally in the mammary fat pads and grown to at least 200 mm^3^. **b** The mice were then randomized across the treatment cohorts. Veliparib (V) (20 mg/kg; low dose (N = 12) or 60 mg/kg; high dose (N = 12) or placebo (N = 9)) was administered per oral gavage three times daily for 3 days (**c**). Carboplatin (C) 60 mg/kg (N = 33) or placebo (N = 6) was administered via intravenous injection on days 1 and 2. Blood samples were taken on day 2 at 0.6, 3 and 5 h after veliparib and carboplatin dosing of 15 mice. **d** In two mice the TNBC tumors were further analyzed using dynamic contrast-enhanced magnetic resonance imaging (DCE-MRI) after the first dose (N = 2). **e** Mice were euthanized on day 3, at 3 h after the last dose of veliparib (N = 39), or at 1.5 h after single-dose V/C (N = 2 (DCR-MRI pilot). Bilateral xenograft tumors and liver and muscle (quadriceps) tissues were obtained. Tissues were divided into three parts and cryo-sectioning was performed in one part. Serial 5-μm-thick or 12-μm-thick sections from each biopsy were cut. H&E staining (**f**), matrix-assisted laser desorption/ionization mass spectrometric imaging (MALDI-MSI) (veliparib) (**h**) and ICP-MS (platinum adducts) (**g**) were performed on subsequent sections of veliparib/carboplatin-treated animals. **i** One part of the tissue was ground for quantification using LC-MS

Forty-one beige DF mice with severe combined immunodeficiency (SCID) (Fox Chase, strain R035948:1) were implanted with 10^6^ MDA-MB-231 (N = 10), HCC70 N = 21) or MDA-MB-436 (N = 10) TNBC cells obtained from the ATCC bilaterally in the mammary fat pads (Fig. 1). When the TNBC xenograft tissue size exceeded 200 mm^3^, the mice were randomized across the treatment cohorts. Animals were randomized into veliparib low dose (20 mg/kg), veliparib high dose (60 mg/kg) or placebo + C, introduced via oral gavage three times daily for 3 days (all but the two DCE-MRI study mice). Carboplatin at 60 mg/kg was administered intravenously on day 1 and day 2. Blood samples (50 μL) were obtained at day 2 in ethylenediaminetetraacetic acid (EDTA) containing vials at 0.6, 3 and 5 h after veliparib and carboplatin dose in 15 mice (all xenograft models, Fig. 1). The procedures were approved by the Institutional Animal (IACUC AN092211-01D).

### Sample preparation and analysis by MALDI-MS, ICP-MS, LC-MS and H&E

Thirty-nine mice were euthanized approximately 3 h after the last dose of veliparib and 24 h after the last dose of carboplatin; two mice used in the DCE-MRI pilot were euthanized 1.5 h after single-dose administration of veliparib and carboplatin (Fig. 1). Bilateral xenograft tumors and liver and muscle (quadriceps) tissues were harvested. Tissues were cut into three pieces and either embedded in optimum cutting temperature (OCT) formulation or were directly snap frozen in liquid-nitrogen-cooled isopentane and stored at − 80 °C until analysis. Serial 5-μm-thick and 12-μm-thick sections were cut and directly applied onto a stainless steel plate for analyses of veliparib (the parent compound not its inactive metabolites such as M8) by MALDI-MSI, transferred to a tube for DNA extraction and platinum adduct quantification by ICP-MS or stained with hematoxylin and eosin (H&E). A separate fresh-frozen portion of each tissue per animal was ground for quantification of veliparib and carboplatin by LC-MS. Method development and details of the techniques are described in Additional file 1a-c.

### Dynamic contrast-enhanced magnetic resonance imaging (DCE-MRI)

To further characterize tumor perfusion/permeability, DCE-MRI was acquired in two HCC70 xenograft tumor-bearing mice after the first 60 mg/kg veliparib and carboplatin administration and animals were sacrificed 1.5 h after veliparib/carboplatin (V/C) administration (Fig. 1) and LC-MS + MALDI analysis of the tumor tissues was performed. Details of the DCE-MRI methods and analyses are described in Additional file 1d.

### Patient sample analysis

Carboplatin and platinum adducts were determined in a limited number of blood samples from a previously presented phase 1 clinical trial (NCT02358200) [28]. In this study, patients treated sequentially with carboplatin single agent at day 1 at a starting AUC of 1.5 mg/ml x min administered weekly and then combined with the PARPi talazoparib (starting at day 2 at a starting dose of 0.75 mg daily). In the case of grade 3 or grade 4 toxicity, carboplatin and/or talazoparib doses were held until below grade 1 and resumed at a lower dose level. Blood samples were obtained from a limited number of patients during cycle 1 day 1 (prior to talazoparib dosing) and cycle 2 day 1 pre-dose and up to 24 h post dose, and PBMCs were derived at day 15 post dose, DNA was extracted as described in Additional file 1d and platinum adducts were quantified by ICP-MS (Additional file 1b).

### Statistical and PK analyses

Data management, analyses and visualization were managed using R (R-3.1.1, Development Core Team (2013)). The paired *t* test and Pearson correlation was used to compare tissue concentrations versus plasma concentrations. The concentration of veliparib assessed by LC-MS and platinum adducts in tumor tissues by ICP-MS were compared among the three TNBC xenograft cell sources using one-way analysis of variance (ANOVA) and correlation with concentrations of veliparib was tested using Pearson correlation. In these analyses no adjustment was performed for multiple comparisons. Given the small size of the study, these statistical calculations are descriptive (e.g. *P* values are measures of distance with no inferential content).

Heterogeneity of drug distribution of veliparib in the tissues evaluated by MALDI-MSI was assessed by comparing the relative standard deviation in the whole tissue of muscle and tumor cells, and by comparing the mean pixel intensity of each immunohistochemically (IHC)-defined region of interest capturing the whole tumor region, and regions of cellularity/necrosis using one-way ANOVA.

The concentrations of veliparib and carboplatin in plasma and tumor quantified by LC-MS and ICP-MS were included in PK analyses to quantify drug penetration. PK-analyses were performed using nonlinear mixed-effects modeling (NONMEM VII Software, ICON Development Solutions, San Antonio, TX, USA), using the first-order conditional estimation with interaction (FOCEI) method. The model-building procedure was guided by the likelihood ratio test, diagnostic plots and internal model validation techniques, including visual predictive checks and bootstrap analysis. The effect of dose, concentration and cell source on TNBC xenograft tissue concentrations was assessed. The variability between animals was assessed using two components: one consistent difference common to all TNBC xenograft tissue samples from left and right mouse tumor and one mouse-specific difference (RRES) to understand the inter-individual and intra-individual variability.

### Spike-in measurements of veliparib penetration in patient tumors

In order to assess any tissue-specific ionization effects for veliparib and estimate the limit of detection of the MALDI-MSI method in different patient tissue/cell types, untreated 5-μm and 12-μm sections of patient tissues of benign tissue with epithelial cells, adipose tissues, breast cancer tumor and stroma tissues were collected from the University of California San Francisco (UCSF) Helen Diller Family Comprehensive Cancer Center Tissue Core and fresh frozen OCT-embedded 9-gauge needle biopsies from the Susan G. Komen Tissue Bank. These tissues were spiked with 1 fmol to 100 pmol absolute drug amount and analyzed using MALDI-MSI.

### Simulation to predict veliparib penetration in patient tumors

The population PK model developed in patients and published by Salem et al. [29] was used to evaluate the plasma PK and predict veliparib plasma concentrations in patients. Scaling tumor concentrations from mice to humans was achieved by linking the mouse tumor model to this previously published plasma PK model in patients [29], as shown in Additional file 1: Figure S11. This linked model was used to simulate concentration-time profiles in tumor and plasma in 1000 hypothetical subjects using the dosage given in the I-SPY 2 trial (50 mg twice daily (BID) for 12 weeks) and the maximum tolerated dose (400 mg BID), assuming perfect drug adherence.

The model assumptions for therapeutic clinical concentrations for veliparib were derived from reported in vitro concentrations that achieved at least 50% reduction in cell growth (IC_50_) in triple negative breast cancer cell lines (TNBC), either as a single agent or in combination with carboplatin as described by Hassan et al. [30] and IC_50_ for PARP1 inhibition of 4.7–5.1 nM [3, 31]. As veliparib has protein binding capacity of 51% in human plasma [32], the obtained IC_50_ values were adjusted for protein binding in human plasma for comparison of equivalent unbound concentrations [33].

## Results

### Xenograft breast cancer models showed a high implantation rate

**Fig. 2:**
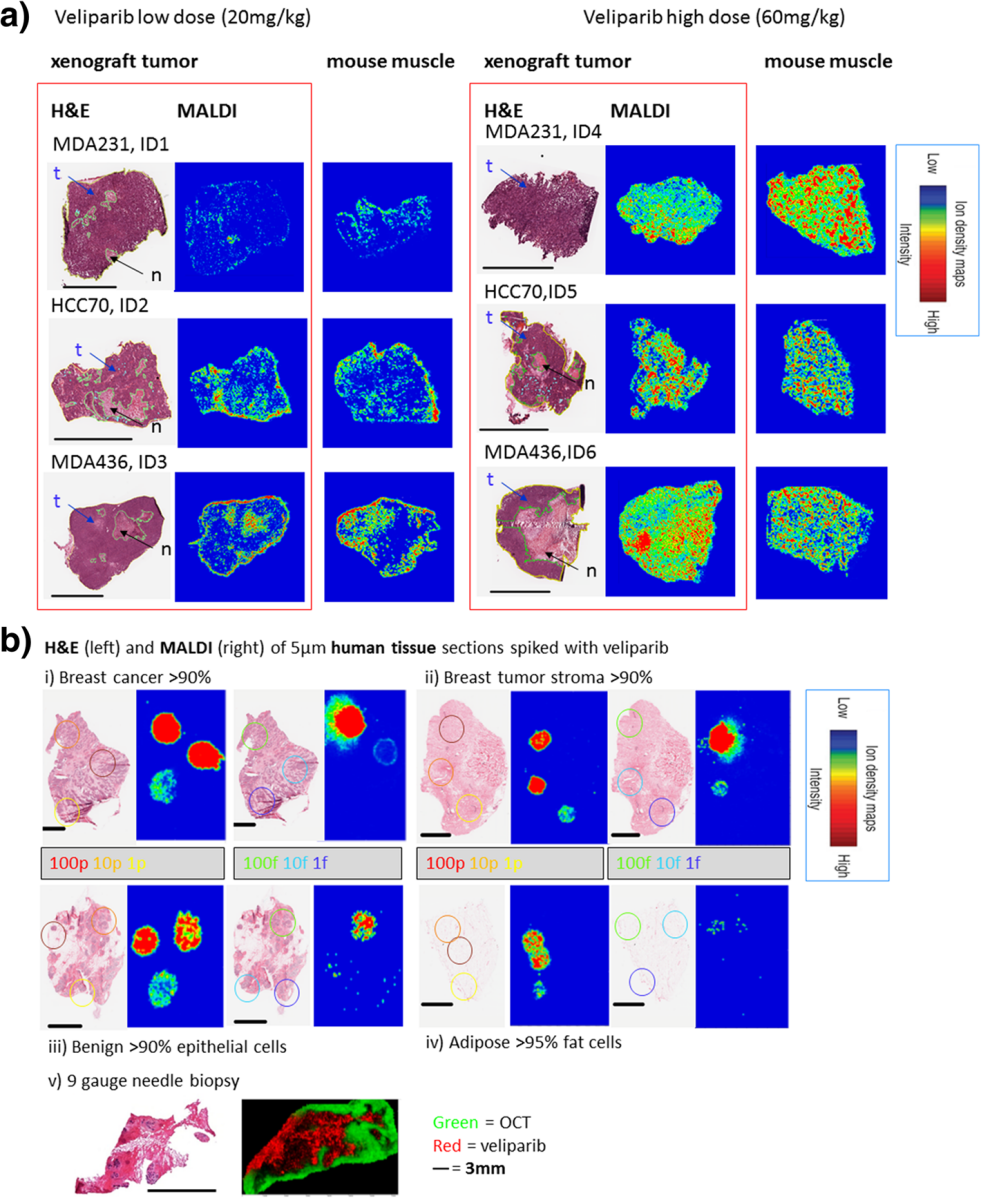
Spatial distribution of veliparib in representative examples (**a**) after low dose (20 mg/kg) and high dose (60 mg/kg) administration using 12-μm-thick tissues from mice xenografted with three triple negative breast cancer cell lines, 3 h after the last dose. H&E (left) and matrix-assisted laser desorption/ionization mass spectrometric imaging (MALDI-MSI) (middle) of veliparib in each xenograft tumor are shown for two mice per cell type. The MALDI-MSI image of the muscle tissue of the same animal (right) is shown for comparison of variability in veliparib distribution between tissues. **b** Shows that veliparib can be measured in breast cancer tissues (i), stroma tissue (ii), benign tissue (iii) and in 9-gauge core biopsies (v), but the limit of detection is much higher in adipose tissues (iv). The 100p-1p and 100f-1f images were generated using separate intensity scales, allowing the lower concentration spots to be visualized. The colors in the MALDI images represent the veliparib concentration, with blue denoting the minimal signal intensity observed and red denoting maximal signal intensity per image. The regions of interest were derived from the delineated areas in the H&E stains. Yellow, total area of tumor; green, area of frank necrosis (and in some cases adjacent non/hypocellular dropout areas); dark blue, area of frank necrosis and adjacent non/hypocellular dropout areas are also present; aqua blue, spot foci of necrosis and/or apoptosis scattered throughout the tumor. *Mice used for the dynamic contrast-enhanced magnetic resonance imaging (DCE-MRI) pilot; further details are shown in Fig. 5. t = viable tumor cells, N = necrotic cells, scale bar = 3 mm in all tissues

The implantation of TNBC cells successfully produced tumors in all 41 SCID mice. H&E stains of tumor tissues showed large numbers of viable tumor cells and necrotic pockets throughout (H&E stains in Fig. 2 left). In particular, MDA-MB-436, a basal-B subtype, BRCA1-mutated cell line [17], produced tumors that had necrotic cores in both placebo and veliparib + carboplatin treated cases. This is consistent with the aggressive nature of these cell types resulting in rapid tumor growth and necrosis.

### Concentration of veliparib differs between tissues

**Fig. 3:**
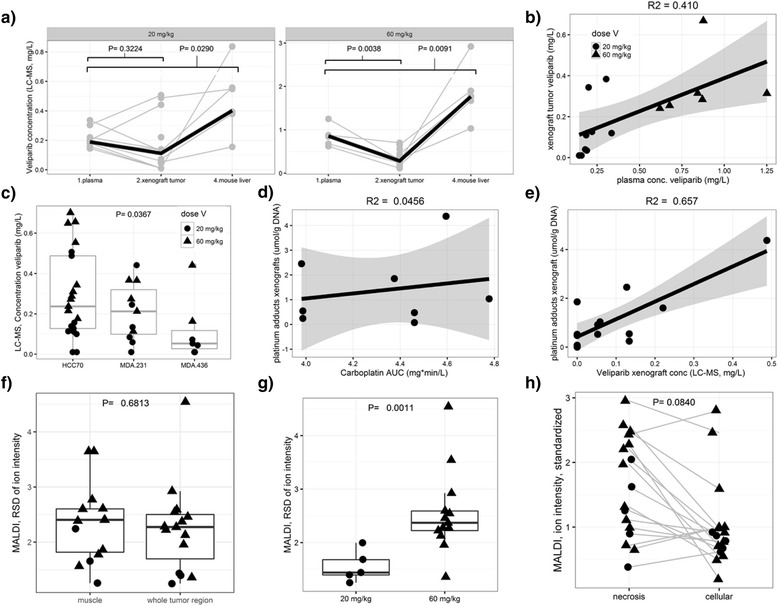
**a** Concentrations veliparib (20 mg/kg and 60 mg/kg three times daily) quantified using LC-MS in plasma, in the three xenograft triple negative breast cancer (TNBC) cell lines and in liver tissues at the time of killing, 3 h post dose. **b** Correlation between veliparib plasma and tumor concentrations at the time of killing, 3 h post dose. **c** Difference in veliparib concentrations among the TNBC xenografts. **d** Correlation between total carboplatin concentrations in plasma (AUC as determined by NONMEM; see Additional file 1: Table S1) and platinum adduct formation determined by ICP-MS in the TNBC xenografts at the time of killing, 3 h post dose. **e** Correlation between veliparib penetration and platinum adduct formation in the TNBC xenografts at the time of killing, 3 h post dose. **f** Comparison of heterogeneity in the distribution of veliparib assessed by matrix-assisted laser desorption/ionization (MALDI) in the tumor region and muscle tissue using the relative standard deviation in pixel intensity as the measurement of spatial heterogeneity. **g** Comparison of heterogeneity in the distribution of veliparib assessed by MALDI after low dose (20 mg/kg) and high dose (60 mg/kg) veliparib administration using the relative standard deviation in pixel intensity in the whole tumor region as the measurement of spatial heterogeneity. **h** Comparison of the spatial distribution of veliparib assessed by MALDI in necrotic versus non necrotic regions. In this analysis, image intensity was standardized over the tumor/muscle ratio to normalize to variability in doses and pharmacokinetics. Data are shown per dose level (circle = 20 mg/kg, triangle = 60 mg/kg dose) and per individual or cell source. The black line is either the median (**a**) or the linear correlation (**b**, **d**, **e**). The *R* ^2^ value when comparing concentrations is provided. AUC area under the concentration-time curve, RSD relative standard deviation

Veliparib was quantified by LC-MS in 36 TNBC xenograft tumors, 11 muscle tissues and 12 liver tissues (Fig. 1). Veliparib and carboplatin plasma levels were measured at three time points after administration in 15 animals (all xenograft models). At 3 h after the last dose, veliparib concentrations in TNBC xenograft tissue were lower than plasma concentrations in all but 2 animals, with 8 of 19 tumor concentrations with low dose veliparib below the limit of detection, while concentrations in the liver were significantly higher (Fig. 3a, Table 1). At the high dose, the mean total veliparib concentration in tumors was 0.36 mg/L (95% CI 0.14–0.68) and in the liver it was 1.77 mg/L (CI 1.11–2.79), versus 0.86 mg/L in plasma (95% CI 0.63–1.20, Fig. 3a, Table 1). TNBC xenograft tissue concentrations correlated with plasma concentrations (*P* = 0.001, *R* ^2^ = 0.41, Fig. 3b). In a multivariate model adjusting for dose level, total veliparib concentrations differed significantly among xenografts derived from TNBC cell lines (*P* = 0.037), with the highest levels found in HCC70 tumors (at the highest dose level, 0.43 mg/L in HCC70 compared to 0.27 mg/L in MDA-MB-231 and 0.32 mg/L in MDA-MB-436, Fig. 3c).

**Table 1:** Veliparib concentrations by LC-MS (left) and its spatial distribution by MALDI (bottom) varies by tissue, drug dose and TNBC cell line of origin

	Tissue	Amount of tissue median (RSE)	Comparison		Method	Subjects (*N*)	Tissues (*N*)	Tissues (*N*) <LOD; <LOQ	Median conc. (RSE %)^a^	Ion intensity tumor/muscle (RSE %);	*P*
a) Veliparib	Plasma	50 μL	20 mg/kg 60 mg/kg		LC-MS	9 6	9 6	1; 0 0; 0	0.19 (10) *0.86 (11)*		<0.001 (Low/high dose)
	Muscle	49.9 mg (13%)	20 mg/kg 60mg/kg			5 6	5 6	2; 3 1; 1	0.12 (70) *0.47 (47)*		0.882 (Muscle/plasma)*
	Liver	49.9 mg (12%)	20 mg/kg 60 mg/kg			6 6	6 6	0; 0 0; 0	0.47 (20) *1.77 (14)*		<0.001 (Liver/plasma)*
	TNBC xenograft tissue	46.1 mg (8%)	20 mg/kg 60 mg/kg			12 12	19 17	8; 15 0; 1	0.12 (31) *0.36 (12)*		0.322 (Tumor/plasma) 0.0038 (tumor/plasma)
			HCC70	20 mg/kg		4	6	2; 4	*0.13 (43)*		0.0367 (Cell source)*
				60 mg/kg		5	9	0; 0	*0.43 (16)*		
			MDA-MB-231	20 mg/kg		4	7	4; 6	0.01 (62)		
				60 mg/kg		4	5	0; 1	0.27 (18)		
			MDA-MB-436	20 mg/kg		4	6	2; 5	0.05 (47)		
				60 mg/kg		3	3	0; 0	0.32 (26)		
		12 μm section	20 mg/kg 60 mg/kg		MALDI	6 9	6 12			0.98 (16); 0.02 *1.36 (19); 0.13*	<0.001 (Low/high dose)
			Necrotic tumors Cellular tumors			15 15	18 18			*1.63 (70%)* 1.01 (85%)	0.084 (ROI)*

*MALDI* matrix-assisted laser desorption/ionization, *TBNC* triple negative breast cancer, *PBMC* peripheral blood mononuclear cells, *RSE* relative standard errors, *ROI* region of interest, *V* veliparib

^a^Concentration (Conc.) of veliparib and carboplatin in mg/L, carboplatin adducts in μmol/g DNA

*P value adjusted for dose

### Platinum adduct formation in xenografts appears to be correlated with veliparib penetration

Carboplatin concentrations in plasma of mice decreased from 24 mg/L (relative standard error (RSE) 20%) to undetectable by LC-MS at 5 h after administration in plasma and was undetectable in the TNBC xenograft tissues 24 h after administration of carboplatin by MALDI-MSI and LC-MS (Table 1b). Platinum adduct formation was quantified by ICP-MS analysis of 13 TNBC xenograft tumors 24 h after two carboplatin administrations, combined with either placebo (N = 5) or veliparib 60mg/kg for 3 days (N = 8). DNA extraction from 12-μm sections resulted in 0.10–9 μg DNA. The median platinum adducts value was 546.3 nmol/g DNA (range 0.2558–4371) in xenograft tumor sections, with all adducts were above the limit of detection in all samples (Table 2). In this small subset, platinum adducts were not significantly different among HCC70, MDA-MB-231 and MDA-MB-436 xenografts (1.85, 0.55 and 0.54 μmol/g DNA, *P* = 0.091 (Table 2). Platinum adduct formation did not correlate with carboplatin plasma exposure (area under the curve (AUC), *R* ^2^ = 0.045, Fig. 3d), but platinum adducts appeared to be correlated with veliparib penetration in the xenograft tumors, though the numbers were small (*R* ^2^ = 0.657, *P* < 0.001) (Fig. 3e).

**Table 2:** The concentration of carboplatin in plasma by LC-MS (left) and in xenograft tissues and patient PBMCs by ICP-MS (bottom)

	Tissue	Amount of tissue median (RSE)	Comparison	Method	Subjects (*N*)	Tissues (*N*)	Tissues (*N*) <LOD; <LOQ	Median conc. (RSE %)^a^	*P*
b) Carboplatin	Plasma	50 μL	0.6 h	LC-MS	15	15	0; 0	24 (20)	
			5 h		15	15	15; 15	<0.01	
Carboplatin adducts	TNBC xenograft tissue	12 μm sections: 1.9 μg DNA (56%)	All	ICP-MS	13	13		0.55 (30%)	
			HCC70		5	5		1.85 (42%)	0.091(Cell source)
			MDA-MB-231		3	3		0.55 (23%)	
			MDA-MB-436		5	5		0.54 (27%)	
			Placebo		5	5		0.476 (57)	0.087 (V yes/no)
			Veliparib 60mg/kg		8	8		0.977 (33)	0.083(Cell source + V)
	PBMCs from patients	Derived from 5–15 mL blood, extracted from a median of 2,160,000 cells			51	51		0.0552 (30%)	

*MALDI* matrix-assisted laser desorption/ionization, *TBNC* triple negative breast cancer, *PBMC* peripheral blood mononuclear cells, *RSE* relative standard errors, *ROI* region of interest, *V* veliparib

^a^Concentration (Conc.) of veliparib and carboplatin in mg/L, carboplatin adducts in μmol/g DNA

*P value adjusted for dose

### PK model parameterized on mice suggests low tumor penetration in TNBC and no interaction between veliparib and carboplatin in plasma

**Fig. 4:**
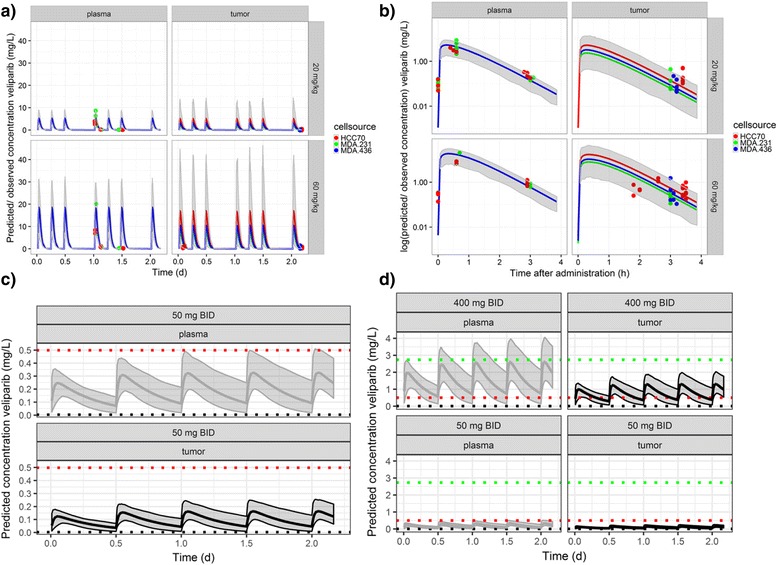
Observed and the associated model-predicted plasma and tumor (LC-MS derived) concentrations at 3 days (**a**) and after the last veliparib administration (**b**) show that the pharmacokinetic (PK) model (shaded area shows 95% CI prediction interval and the straight line shows the typical PK profile) of the xenograft mouse data is able to reproduce the central tendency in the observed plasma and xenograft veliparib concentrations (dots, colored by TNBC xenograft cell line). **c** Simulation of plasma and tumor concentrations in patients show that plasma and tumor exposures in the mice treated with 20 mg/kg veliparib are similar to patient exposures after 50 mg twice daily (BID) dosing. The line represents the typical PK profile and shaded areas are the simulated median with 95% CI uncertainty of the simulated concentrations in plasma (gray) and tumor (black). **d** Comparison of the simulated concentrations with in vitro derived half maximal inhibitory concentration (IC_50_) values observed in breast cancer cells suggests that concentrations at steady state of veliparib 400 mg BID may be sufficient for patients with a somatic or germline BRCA mutation (using IC_50_ values observed in BRCA mutated breast cancer cells, red dotted line), but may be below the effective concentration in non-BRCA carriers (using IC_50_ values observed in non-BRCA mutated breast cancer cells, green dotted line)

In order to estimate the veliparib tumor penetration and between-subject and within-subject variability in mice, and predict its behavior in patients with TNBC, the PK of veliparib was parameterized, using the concentrations of veliparib in plasma and tumor quantified by LC-MS as described previously. The time-plasma concentration data for veliparib indicated that the plasma PK in mice displayed non-linear clearance. Such clearance behavior resulted in prolonged high plasma levels at high doses, with a KM of 26.2 mg/L (RSE 57%) and Vmax of 1.86 ml/h (RSE 46%, Fig. 4a (right), Additional file 1: Table S1).

A linear model with immediate equilibrium between the plasma and tumor compartment (Eq. 1) best described the drug penetration in the TNBC xenograft tumor tissue (Fig. 4a (left), Additional file 1: Table S1):

1 $$ \frac{{\mathrm{dC}}_{\mathrm{tumor}}}{\mathrm{dt}}=\mathrm{KPT}\times \left(\mathrm{RPT}\times {\mathrm{C}}_{\mathrm{plasma}}-{\mathrm{C}}_{\mathrm{tumor}}\right) $$

Here KPT describes the rate of transfer from plasma to tumor and RPT describes the ratio of veliparib between plasma and tumor cells. The concentration of veliparib in plasma, not the dose of veliparib administered, appeared to be the driver of drug penetration in the xenograft tumor difference in objective function (ΔOFV) − 513.3, *P* < 0.0001) and an immediate equilibrium between plasma and tumor compartment was estimated (KPT = 900 ml/h). Drug penetration from plasma to xenograft tumor was low and dependent on the source of the tumor cells (ΔOFV − 77.2, *P* < 0.001); penetration was 35% (RSE 13%) in MDA-MB-231, 74% (RSE 23%) in HCC70 and 46% (RSE 40%) in MDA-MB-436 derived xenografts (Fig. **4**b). Plasma concentrations and cell source explained 72% and 4% of the observed variability, respectively. Variability within the tumor sections of an animal was 28%, whereas variability between tumors in different animals was 37% (Additional file 1: Table S1a).

The PK of carboplatin in mice was assessed using a one-compartment model with bolus intravenous injection. The half-life of carboplatin in beige SCID mice was 0.35 h, leading to a median area under the concentration-time curve (AUC) of 4.2 mg/min/L in 2 days, RSE 1.3%, (Additional file 1: Table S1b).). No drug−drug interaction was observed between veliparib and carboplatin concentrations in plasma: clearance and volume of distribution of carboplatin were not significantly different with the 0, 20 or 60 mg/kg veliparib doses (ΔOFV − 0.4, *P* = 0.53 versus ΔOFV − 0.6, *P* = 0.43).

### Platinum adducts in PMBCs in patients may correlate with gBRCA status

Full PK profiles of carboplatin in plasma were obtained in the first three patients in the trial, with a total of 51 plasma samples. Platinum adducts was determined in 52 PMBC samples from 19 patients in a previously presented phase 1 clinical trial [28]. The median platinum adducts value was 55.2 nmol/g DNA equivalent to 81.4 pg/L blood, (range 0.009–4641 pg/L) in patient PBMC samples and was detectable up to 15 days post dose, in contrast to the faster clearance of carboplatin in plasma, which was eliminated within h (Additional file 1: Figure S12, half-life carboplatin in plasma = 2.1 h). There was a significant amount of platinum adduct at baseline in the PMBCs from patients who received platinum more than 5 weeks prior to the current treatment: median 0.325 pg/L (range 0.007–2.74 pg/L) in patients without prior platinum treatment versus 4.935 pg/L (range 0.091–867) in patients with prior platinum treatment, *p* value = 0.007898, *p* value = 0.007898 (Additional file 1: Figure S13).

A three-compartment model best described carboplatin in plasma and no variability between patients was observed (Additional file 1: Figure S12 and Table S2). In further analyses, we assumed that the carboplatin plasma PK profile in the other 16 patients was similar to that the first 3 patients: in these patients glomerular filtration rate (GFR) ranged from 60 to 151 mL/min and BSA values ranged from 1.58 to 2.0 m^2^. Correlation between GFR and carboplatin PK was not tested as a prior study showed that GFR was not related to carboplatin clearance in patients with GFR > 50 mL/min. [34].

**Fig. 5:**
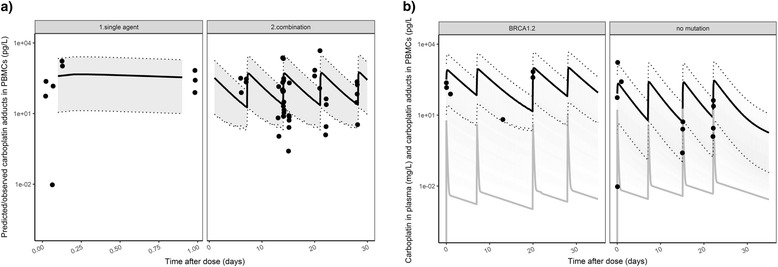
Visual predictive check (VPC) of platinum adducts observed (dots) and predicted (shaded area shows 95% CI prediction interval and the straight line the typical pharmacokinetic profile) in peripheral blood mononuclear cells (PBMCs) from patients stratified for treatment with single agent (**a**) at day 1 (left) and combined with a poly(ADP-ribose) polymerase inhibitor (PARPi) (talazoparib (right)), suggesting that platinum adduct formation was not influenced by the PARPi. **b** VPC stratified for gBRCA patients and non-carriers, showing the results for two individuals. These plots suggest that lower carboplatin adduct formation was observed in patients with BRCA 1/2 mutations (left) compared to non-carriers (right)

The platinum adduct formation in PBMCs in 19 patients was linked to carboplatin concentrations in plasma using the Eq 1. A slow formation rate of adducts from plasma (KPT = 0.0156 L/h) and a small ratio of adducts compared to plasma concentration (RPT = 0.5%) was estimated. In this model, we tested the hypothesis that PARPi interact with platinum formation. In contrast to the data observed in mice tumors, no statistical difference was observed in adduct formation between single-agent treatment at day 1 and combination treatment (Fig. 5a and Additional file 1: Figure S14). The formation of adducts correlated with the number of lymphocytes (*P* < 0.0001) and the gBRCA mutation status (*P* < 0.0001). The percentage of platinum adducts was significantly higher in five gBRCA carriers (ratio of adducts in PBMCs/plasma concentration 0.64% (IQR 0.60–1.16%)) versus nine non-carriers (ratio 0.29% (IQR 0.21–0.66%, *P <* 0.0001, Fig. 5b).

### Imaging veliparib distribution with MALDI-MSI in mouse and patient tissues

The MALDI-MSI method produced high-quality MS images showing the distribution of veliparib in xenograft tumor tissue and muscle in SCID mice with a 20 mg/kg (N = 6) and 60 mg/kg dose (N = 12) (Fig. 2a and Additional file 1: Figure S6).

### Spatial differences in veliparib in TNBC tissues as a function of dose and xenograft type

Visual inspection of the 18 MALDI-MSI images of TNBC xenograft tumor tissues in SCID mice showed a dose dependence of veliparib penetration into the tumor (low dose veliparib versus high dose veliparib (Fig. 2)), heterogeneous drug distribution within all individual tumors (Additional file 1: Figure S6) and some intra-individual variability in two bilateral tumors obtained from the same mouse (Additional file 1: Figure S7a). To quantify these observations, we calculated the mean pixel intensity and the RSD in each IHC-defined region of interest in tissues capturing the whole tumor region, the muscle (without adipose tissue) and regions of cellularity/necrosis specifically (Additional file 1: Figure S6). Veliparib penetration in xenograft tumors was highly heterogeneous (RSD in the tumor region = 224%, 95% CI 129–397%), but was similar to the heterogeneity in non-tumor tissue (RSD in muscle was 236%, 95% CI 136–364%, Fig. 3f). Veliparib penetration in xenograft tumor cells differed between the two dose levels (*P* < 0.001, Table 1) and the observed heterogeneity (assessed using the RSD of the pixel intensity in the whole tumor region) was larger with the 60 mg/kg dose (RSD 155% versus 255%, *P* = 0.001, Fig. 3g). We observed veliparib penetration in necrotic tissue and non-necrotic tissues and the mean pixel intensity in the two regions was not significantly different between the two regions (*P* = 0.084, Fig. 3h, Table 1). In addition, we observed accumulation of veliparib in adipose tissue in some samples (examples are shown in Additional file 1: Figure S7b).

### Extrapolated patient veliparib PK profiles predict similar concentrations in mice and patients

To estimate the limit of detection of veliparib by MALDI-MSI in patients, veliparib was analyzed by MALDI-MSI after spiking decreasing concentrations of the drug onto four different patient tissues containing epithelial cells, adipose tissues, breast cancer tumor and stromal tissue [35]. No interfering background signals were observed. The estimated detection range of veliparib in human tissues was between 10 fmol and 100 pmol absolute drug (Fig. 2b). In adipose tissue, the limit of detection was higher (10 pmol). In 9-gauge biopsies (obtained from Susan Komen Tissue Bank), veliparib was detectable and OCT-embedding only dampened the signal 0.2 mm from the edge of the tissue in the core biopsy (Fig. 2b, bottom).

PK parameters derived from the literature for veliparib exposure in patient plasma were linked to simulate and compare plasma concentrations in the patients (Additional file 1: Figure S11). Clinical trial simulations of veliparib at 50 mg BID dose suggest that plasma exposure and tumor exposure in patients from the I-SPY 2 trial were similar to the concentrations in mice dosed at 20 mg/kg, falling within the established limits of detection of the MALDI-MSI method (Fig. 4c). When concentration were compared to IC_50_ values derived in vitro, clinical trial simulations predicted that at full adherence BRCA mutation carriers reached the IC_50_ for PARP1 inhibition, but tumor concentrations did not reach the IC_50_ in the cell proliferation assays as threshold of response patient simulations (n = 1000, Fig. 4d). Figure 4e shows that concentrations at the steady state of veliparib 400 mg BID may be sufficient for patients with a somatic or germline BRCA mutation, but may be below the effective concentration in non-BRCA carriers (using IC_50_ values observed in these breast cancer cells in vitro) [30].

### Example images comparing DCE-MRI features to MALDI-MSI drug uptake images

**Fig. 6:**
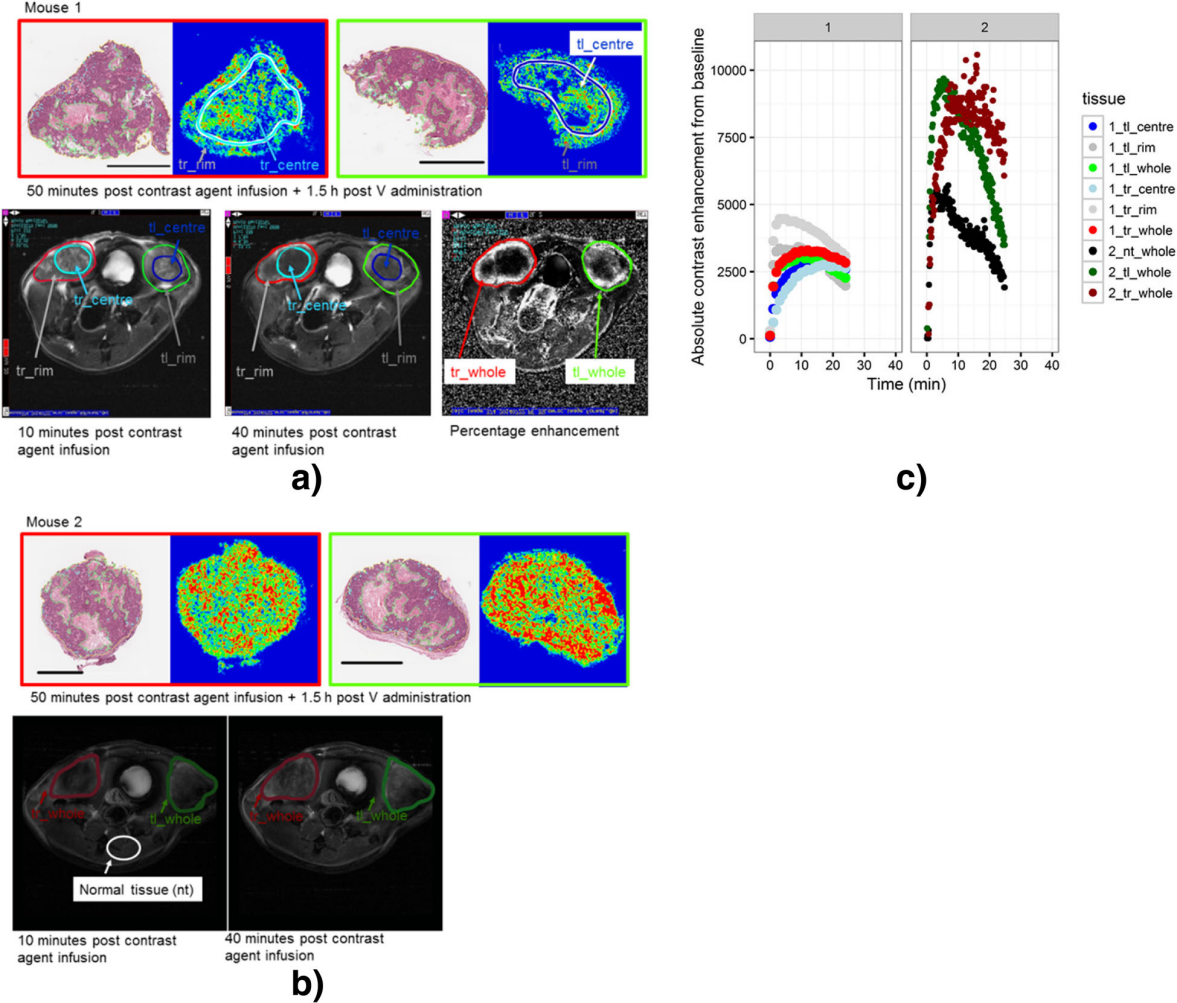
Visual comparison of H&E stains and veliparib penetration. Matrix-assisted laser desorption/ionization mass spectrometric imaging (MALDI-MSI) and dynamic contrast-enhanced magnetic resonance imaging (DCE-MRI) images of two HCC70 mice xenografts treated with single-dose 60 mg/kg veliparib + 60 mg/kg carboplatin and euthanized 1.5 h after veliparib/carboplatin (V/C) dosing (**a**, **b**). Two implanted xenograft tumors were visualized per mouse. The tumors implanted in the right fat pad is shown in red and in the left fat pad in green (center of the tumor in blue). H&E stains and veliparib MALDI-MSI of the right (red outline) and left (green outline) tumors are shown. DCE-MRI image obtained 10 minutes after infusion of the contrast agent (left) and at 40 minutes (middle and the initial peak enhancement (PE)) (right, mouse 1 only). **c** The signal intensity-time curves during DCE-MRI after the administration of contrast agent in the two HCC70 mice xenografts (left and right panel) of the right (red) and left (green) tumors (rim in grAy and center in blue), and normal tissue (black). The delineated areas in the H&E stains are: yellow, total area of tumor; green, area of frank necrosis (and in some cases adjacent non/hypo-cellular dropout areas); aqua blue, spot foci of necrosis and/or apoptosis scattered throughout the tumor. nt = normal tissue, tr = tumor right, tl = tumor left

With the hypothesis that PK parameters derived from DCE-MRI reflect local blood supply and correlate with drug penetration of diffusible hydrophilic compounds (that can cross the endothelium of capillaries) in tissues, we performed a pilot study in which DCE-MRI was combined with MALDI-MSI in two mice bearing HCC70 xenografts. This cell line was selected due to its tendency toward rapid growth without a large necrotic core in the absence of drug (due to tumor growth outpacing vasculature, not the veliparib effect). The MRI images at 10 minutes showed rim enhancement in the xenograft tumor due to the presence of contrast agent (peak enhancement (PE) rim = 1.22 versus PE center = 1.03, Fig. 6a). At 40 minutes the MR images show areas of contrast agent enhancement throughout the xenograft at locations corresponding to the necrotic regions also seen in the H&E-stained tumor sections from the same tumor level (Fig. 6b and c). The MALDI-MSI data support the MRI data in that by the later time point (40–90 minutes) veliparib was distributed throughout the TNBC xenograft in agreement with the contrast agent distribution.

## Discussion

Extensive knowledge of the tumor and its microenvironment suggests that penetration of small molecules may be limited in some solid tumors, and preclinical studies show heterogeneous penetration of cancer treatments in tumors and other tissues [7, 14, 36–38]. However, assessment of spatial drug distribution in human tumor tissues has been hampered by technical challenges and clinical assessment of drug penetration has been limited to fluorescent-labeled or radio-labeled drugs [39]. Platinum adducts have been measured in tumors and PBMCs, and these measurements have been successfully related to outcomes [12, 19, 40], but using prior MS methods, at least 1 mg DNA was needed [12, 18, 41]. In our study immunofluorescence techniques to detect carboplatin adducts have successfully been used on a cellular level, but quantification of the fluorescence signal is not straightforward, therefore comparison between samples and studies was limited [40, 42, 43]. Platinum adducts were quantifiable in small amounts (0.10–9 μg) of DNA in tumors and PMBCs from patients 15 days post treatment. In addition, the presented MALDI-MSI approach produced high-quality images of drug distribution, using concentrations predicted to be observed in patients with TNBC. These results demonstrate the potential to use MALDI-MSI and ICP-MS to assess the distribution of PARPi and platinum adducts in clinical samples (tumor sections from large core needle biopsies).

Although susceptibility of the tumor to DNA repair insults is key in the understanding the response to PARPi, the results of our study suggest that limited drug penetration into the target lesion may account for some level of non-response. Patients with a tumor type known to have homologous recombination repair defects, such as those arising in patients with mutations in BRCA 1/2 germline or DNA-damage repair-related genes (e.g. BRCAness or mutations in DNA damage sensors ATM/ATR or PTEN), are most likely to benefit from PARPis given alone or in combination with DNA-damaging agents (the concept of synthetic lethality) [44]. This is supported by our observation that at maximum tolerated dose, veliparib may not reach effective concentrations in non-BRCA carriers (using IC_50_ values observed in breast cancer cells in vitro) [30]. But in tumors susceptible to DNA repair insults, sufficient concentrations to inhibit PARP enzymes are still needed. Specifically at the 20 mg/kg dose - equivalent to the concentrations predicted using the 50 mg BID dose in patients - 42% of the xenograft tissues were below the limit of detection by LC-MS - below the IC_50_ values observed in gBRCA mutated cells. Drug penetration was increased at the higher dose, but increased spatial heterogeneity of the veliparib distribution was also observed. This may imply that even when increasing the dose, some tumor areas may not receive the required therapeutic level at some time during the dosing interval.

The ability to measure the penetration of a PARPi and DNA-damaging agent in tumors as biomarkers of efficacy is especially relevant for PARPi due to its mechanism of action. Poly(ADPribosyl)ation, catalazed by PARP, is a crucial part of the DNA damage response system for sensing DNA lesions, activating DNA damage response pathways and facilitating DNA damage repair [45]. The normal level of poly ADP-ribosylation is very low. At low doses of veliparib (10–50 mg), significant inhibition of PARP levels have been observed in patients in both tumor tissue and in PBMCs [46]. Specifically, in a phase 2 study of patients with advanced solid tumors, veliparib 10–40 mg BID combined with irinotecan 100 mg/m^2^ reduced tumor poly(ADP-ribose) (PAR) content in all tumor biopsies taken from patients 4 h after the morning dose (approximately at Cmax level), but PAR levels were variable and remained above the limit of detection in most samples [47]. Following genotoxic stress (e.g. induced by chemotherapy), the level of poly(ADPribosyl)ation increases 10-fold to 1000-fold in a few seconds [45]. Therefore, it is likely that unless PARP1 activity is virtually completely inhibited during the dosing interval, single-strand breaks will largely be repaired before the cell reaches the S-phase [48]. In addition to PARP inhibition, PARP inhibitors may also trap PARP1 and PARP2 on damaged DNA by way of a poisonous allosteric effect [49, 50]. Trapped PARP-DNA complexes may prevent DNA replication and transcription, killing cancer cells more effectively than catalytic inhibition. However, the capacity to trap PARP varies significantly among PARP inhibitors, with limited trapping activity estimated for veliparib: the PARP trapping IC_50_ at 57.4 umol/L^50^ is far above the tumor concentrations measured in our study. Furthermore, BRCA1 protein levels may vary among patients and BRCA1 protein levels inversely correlate with PARP inhibitory activity [51]. In future studies, the correlation between veliparib tumor concentrations, biomarkers of DNA damage repair such as PARP inhibition, PARP trapping and BRCA1 protein expression, and intrinsic “DNA-damage repair deficiency” should be considered in conjunction and the relative contribution of each biomarker to predict treatment response should be assessed.

Although PARPi combined with a DNA damaging agent is a promising approach in BRCA-mutated breast cancer and TNBC, responses are variable between patients, which cannot be attributed only to differences in susceptibility of the tumor to repair DNA repair insults. In a study in metastatic BRCA-mutated breast cancer, in which patients received veliparib 400 mg BID until disease progression, only 20% showed partial response [52]. In the BROCADE 2 study patients with locally advanced or metastatic BRCA-mutant breast cancer were treated with carboplatin/paclitaxel and veliparib 120 mg day 1-7 per 3-weekly cycle (Q3W), at 30% of the maximum tolerated dose, or carboplatin/paclitaxel and placebo [53]. This study showed a trend but did not meet its significance cut off when evaluating progression-free survival benefit [53]. The subsequent BROCADE 3 study (NCT02163694) evaluating standard chemotherapy, single-agent veliparib versus veliparib in combination with carboplatin and paclitaxel, is still enrolling. The phase 3 study of the PARPi olaparib monotherapy provided evidence of statistically significant and clinically meaningful progression-free survival benefit in human epidermal growth factor receptor 2 (HER2)-negative, gBRCA-mutated breast cancer, compared to treatment of the physician’s choice [54]. In addition, veliparib 50 mg BID combined with carboplatin showed significant efficacy in TNBC in the I-SPY 2 trial, which included only three patients with a BRCA-mutation who were on neoadjuvant treatment in the I-SPY 2 trial [4]. In this study approximately 42% of TN patients did not have pathologic complete response (pCR) to veliparib-based treatment [4]. But in phase 3, the addition of veliparib 50 mg PO BID compared to placebo added to neoadjuvant carboplatin (AUC 6 mg/mL/min q3 weeks) and paclitaxel followed by doxorubicin + cyclophosphamide, did not have an increased pCR rate in the breast and nodes in stage II–III TNBC [55]. Based on the observed variability in responses in breast cancer irrespective of mutations in DNA damage repair we can extend the hypothesis that insufficient or heterogeneous veliparib penetration and platinum adduct formation in solid tumors may lead to inadequate response to combination therapy across tumor types.

By inhibition of PARP1 activity, a PARPi slows down the nucleotide excision repair, thereby decreasing the ability to remove inter-strand and intra-strand platinum adducts. Platinum adducts may therefore serve as a biomarker of efficacy of the PARPi/carboplatin combination. In our study veliparib administration did not influence carboplatin exposure in plasma, but in a small number of mouse xenografts we observed increased adduct formation. This potential intracellular drug–drug interaction in mouse xenografts may be a result of the synergistic effects between these two agents and is in line with the results of Olaussen et al., who showed in vitro that platinum adduct formation increased after concomitant administration of a PARPi [56]. In this small analysis of the phase 1 study of carboplatin in combination with the PARPi talazoparib, this drug–drug interaction was not observed in PMBCs. In PBMCs of patient gBRCA1/2 mutations, we observed increased platinum adduct formation in PBMCs, suggesting that these patients may have lower ability to remove inter-strand and intra-strand platinum adducts. As our sample size was small these findings warrant further study of platinum adduct formation and drug penetration in tumor cells.

The concentrations of veliparib in implanted TNBC xenograft tumors were 10-fold lower than the concentrations previously observed after a single dose of veliparib in a melanoma subcutaneous flank model [57]. In our study only 35–74% of veliparib transferred from plasma to TNBC xenografts. This TNBC tumor/plasma ratio was similar to veliparib tissue/plasma ratios previously observed in less perfused tissues such as bone, eye and brain in rats [58]. Veliparib concentrations in the highly vascularized liver tissues was higher, an effect previously also observed in a radioactivity study of veliparib in rats, with renal clearance accounting for 70% of veliparib elimination [46]. Potentially, drug transporters may play a role in limiting drug penetration into some tumor tissues: efflux transporters ABCG2 (BRCP), ABCC2 (MRP2), ABCC4 (MRP4) [59] and ABCB1 (P-glycoprotein) are overexpressed in MDA-MB-436 and MDA-MB-231 [60], whereas uptake transporters OCT1 and OCT2 are highly expressed in HCC70 and MDA-MB-231, but not in MDA-MB-436 [61]. As veliparib is a potential target for drug transporters such as ABCB1, OCT1-3, OAT1-3 and MATE1 ABCG2 [62–64], these observations are consistent with higher veliparib concentrations observed in the HCC70-derived tumors. Low perfusion into poorly vascularized tumor may have prevented penetration of the PARPi into the tumor [10]. Confirming previous studies [13, 35, 65], veliparib, a small, hydrophilic compound with low protein binding diffused well from the cellular rim into the necrotic core of tumors. A pronounced rim enhancement was shown in DCE-MRI immediately post injection of contrast agent, i.e. more pronounced contrast enhancement at the tumor periphery compared to that at the center in these tumors. The early rim enhancement observed in this pilot study may reflect high vascularization at the rim due to aggressive growth and low vascularization of the tumor center, characteristic of TNBC [66]. Rim contrast enhancement may explain low veliparib penetration in implanted TNBC xenografts and has been associated with death and disease recurrence in TNBC [67, 68].

The clinical implications of studying heterogeneity in drug penetration using a single biopsy may be most relevant in the neoadjuvant setting, where limited drug penetration into the target lesion may account for some level of non-responses observed after PARPi/carboplatin treatment. In the case of minimal residual disease, penetration into the tumor core may be less relevant. The single 2D plot MALDI-MSI images shown in this study are representative only of the immediate tissue surroundings of a small target lesion. Due to the highly heterogeneous morphology of the tumors, a semiquantitative readout from a single 2D tissue section is unlikely to represent drug distribution within the entire tumor of 1 cm or greater in diameter (comprising necrotic, cellular and potentially, areas of adipose tissue). Drug penetration may be more heterogeneous in larger tumors and even more across the different tumor sites in metastatic breast cancer. Collection of multiple sites or multiple sections throughout large tumors would provide a more comprehensive understanding of drug distribution, but would substantially increase the MSI sample load. Therefore, we suggest combining MALDI-measured drug distribution from core needle biopsies in a small portion of the tumor with imaging techniques such as DCE-MRI to visualize and correlate drug localization with measurements of tumor perfusion [69]. Another possibility is to combine MALDI-MSI imaging of drug penetration in small molecules at microscopic level with molecular imaging techniques using positon emission tomography (PET) or single-photon emission computed tomography (SPECT) to determine drug penetration in all lesions in the body at a macroscopic level. Molecular imaging probes to visualize PARP1 binding capacity by PET are currently in development [70]. Another caveat of this study is the small number of animals and patients included and the small number of tumor PK samples. Denser sampling in the tumor at multiple time points after drug administrations would improve our understanding of spatial penetration of the drug in the tumor throughout the sampled timeframe. From this initial study, we can conclude that IPC-MS and LC-MS provide fully quantitative data and MALDI-MSI enables detailed spatial information on carboplatin and veliparib uptake into tumors. Future experiments will be targeted to quantify and localize drug distribution by MALDI-MSI by normalization to a stably labeled veliparib internal standard to further enhance the quantitative capabilities of the analysis, and validation by comparison to quantitative LCMS data from laser microdissected areas collected from sections adjacent to MALDI-MSI and H&E tissue sections.

Genomics has enabled the development of targeted therapies. Identification of TNBC subtypes such as lymphocyte predominant and luminal androgen receptor yields promise for personalized medicine in this aggressive type of breast cancer. The spatial, phenotypical and genetic composition of breast cancer and the changes in these in response to therapy are topics of current research [71, 72]. The interaction of genomic instability within the tumor and selective pressures such as differences in tumor micro-environment, changes in endocrine stimuli, and drug treatment may result in intra-tumor heterogeneity in treatment response. The spatial heterogeneity in drug concentrations observed in our study means that there are sanctuary sites that are not or only partially penetrated by drugs. Low concentration in some tumor cells due to heterogeneous drug penetration may cause drug-resistance and treatment failure [73]. For this reason, we suggest that the spatial distribution of drugs should be considered a potential consequence of heterogeneity in tumor composition that impacts the response to drug therapy and shapes the composition of residual disease. By studying the penetration of drugs in tumor biopsies, it may become possible to personalize dosing regimens to improve efficacy and reduce the risk of disease recurrence.

## Conclusions

This (pre)clinical study shows that MALDI-MSI and ICP-MS can be used to measure the penetration of PARPi/platinum in TNBC and that it is relevant, as drug penetration appears to be highly heterogeneous, which may potentially lead to insufficient drug exposure to select cell populations.

## Additional file

Additional file 1: Technical aspects of the MALDI-MSI, ICP-MS and LC-MS methods. Technical aspects of the MALDI-MSI, ICP-MS, LC-MS, DCE-MRI method development and supplements on the PK model development. (PDF 1631 kb)

## Abbreviations

ANOVA: Analysis of variance
AUC: Area under the concentration-time curve (AUC)
BID: Twice daily
DCE-MRI: Dynamic contrast-enhanced magnetic resonance imaging
EDTA: Ethylenediaminetetraacetic acid
gBRCA: Germline BRCA1/2
GFR: Glomerular filtration rate
H&E: Hematoxylin and eosin
IACUC: Institutional Animal Care and Use Committee
IC_50_: Half maximal inhibitory concentration
ICP-MS: Inductively coupled plasma–mass spectrometry
IHC: Immunohistochemical
KPT: Rate of transfer from plasma to tumor
LC-MS: Liquid chromatography–mass spectrometry
MALDI-MSI: Matrix-assisted laser desorption/ionization mass spectrometric imaging
NONMEM: Nonlinear mixed-effects modeling
OCT: Optimum cutting temperature
OFV: Objective function
PARPi: Poly(ADP-ribose) polymerase inhibitors
PBMCs: Peripheral blood mononuclear cells
pCR: Pathologic complete response
PE: Peak enhancement
PK: Pharmacokinetic
RPT: Ratio of veliparib between plasma and tumor cells
RSD: Relative standard deviation
RSE: Relative standard errors
SCID: Severe combined immunodeficiency (SCID)
TNBC: Triple negative breast cancer
V/C: Veliparib/carboplatin
