# Supplementary material for: Heterogeneous drug penetrance of veliparib and carboplatin measured in triple negative breast tumors
Source: Breast Cancer Res. 2017 Sep 11;19:107. doi: 10.1186/s13058-017-0896-4 (PMC5594551; doi:10.1186/s13058-017-0896-4)
Supplement: Additional file 1: — Technical aspects of the MALDI-MSI, ICP-MS and LC-MS methods. Technical aspects of the MALDI-MSI, ICP-MS, LC-MS, DCE-MRI method development and supplements on the PK model development. (PDF 1631 kb) [file 13058_2017_896_MOESM1_ESM.pdf]

Supplement: Technical aspects of the MALDI-MSI, ICP-MS and LC-MS methods

a) Technical aspects of the MALDI-MSI method development.

Sample preparation:

Serial 12 µm thick sections from each tissue sample were directly cut onto stainless steel target plates (Thermo Fisher, San Jose, USA) using a cryotome (Leica Microsystems, Wetzlar, Germany) and 2,5-Dihydroxybenzoic acid matrix (25mg/mL in 50% Methanol 0.1%TFA) was manually applied to the tissue surface using airspray deposition. The airbrush (Paache, Chicago, IL) was positioned at a distance of 30 cm from the tissue and 20 passes over the tissue were performed with the tissue being allowed to dry for 30 seconds between coatings. Iniparib (50pmol/µL Sigma-Aldrich, Milwaukee) was added to the matrix solution as an internal standard.

Data-analysis:

Data-analysis was performed using a MALDI-MSI LTQ Orbitrap XL® mass spectrometer fitted with a 60Hz N2 laser (Thermo Fisher Scientific, Bremen, Germany) at a resolution of 60,000 at *m/z* 400, full width half maximum. The identity of veliparib $[M+H]^+$ at *m/z* 245.141 was confirmed by MS/MS spectra acquired directly from spiked tissue sections (Figure S1). Spectra were acquired in positive ionization mode with a mass window of *m/z* 200-500. Laser energy of 7µJ was applied and 50 laser shots were fired at each position (total of 1 microscan per position). Images were acquired at 50 µm pixel size at which detailed distributions of veliparib within tumor compartments could be visualized. Tissues varied in size between 1-2.5 cm in diameter and acquisition times were between 6 and 19 hours depending upon the size of the tumor tissue.

Figure S1: Graph of Mass spectrometric profile of veliparib and iniparib (internal standard)by MALDI-MS

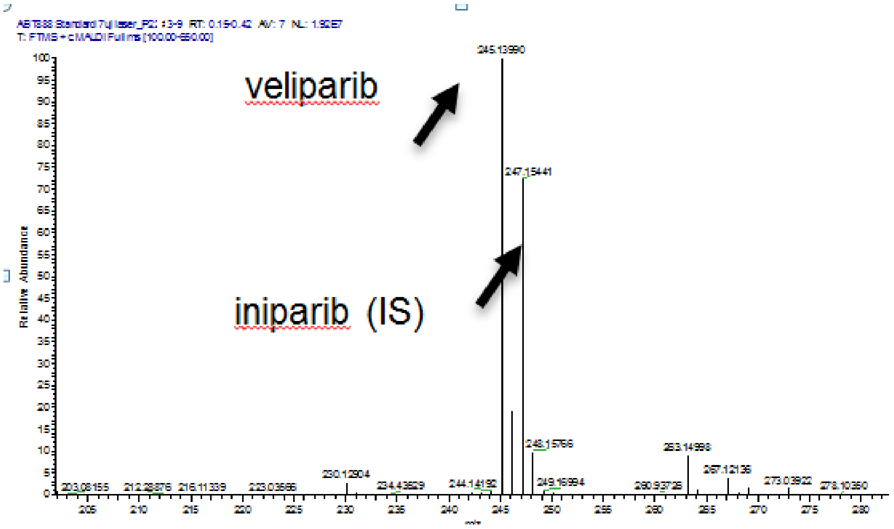

MALDI-MSI data visualization:

Visualisation was performed using Thermo ImageQuest software (v 1.0.1). Normalized ion images of veliparib were generated by dividing the veliparib $[M+H]^+$ signal (*m/z* 245.141 ±0.003) by the iniparib internal standard within the ImageQuest software. Images were displayed in greyscale or 'heat map' color intensity scale. Relative quantitation of veliparib within necrotic, tumor-cellular and central tumor regions was performed using ImaBiotech Software

Quantinetix™(v 1.7, Loos , France). ROIs were drawn by superimposing the MS image over the optical san of the tissue and referring to the H&E mask as a guide.* The mean intensity and standard deviation of the MALDI-MSI signal in ROIs was calculated in the Quantinetix software and exported into R, a statistical analysis software package (version 3.1.2, R Development Core Team (2013, http://www.R-project.org/).

In order to eliminate pharmacokinetic variability due to differences in plasma exposure and compare drug uptake between animals in analysis of the MALDI-MSI data, mean drug signal in the tumor (and subcompartments) were divided by the average drug penetration in the muscle from the same animal, as previously described. [1,2]

*Tumor H&E images were captured using an Aperioslide scanner (Aperio® Leica). Image Scope was used to interpret the H&E stains. Regions of interest (ROIs) were manually drawn to select the whole tumor regions, non-shredded and shredded necrotic and non-necrotic regions.

Validation/method development:

By spiking different concentrations of veliparib on placebo treated mouse TNBC xenograft tissues, a rough estimate of the the detection range of veliparib was determined to be 100 fmol-100 pmol absolute drug amount, Figure S2 left) with saturation of the signal occurring above 1 nmol/ µL. MALDI-MSI Pixel intensity of veliparib in fresh frozen and OCT embedded tissues were compared (Figure S2). In the patient tissues (Figure 2d main manuscript), we were able to detect down to 10fmol in breast stroma and breast cancer tissues. Due to the OCT embedding, we were able to prepare thinner sections (5 instead of 12 µm), which may have improved the LOD. It is the clinical LOD that puts us within the expected detection range for dosed patients. The drug was also detectable in tissue sections mounted on glass slides, but the sensitivity was five-fold lower (data not shown).

Figure S2 Concentrations were spiked on fresh frozen and OCT embedded 12 µm TNBC sections from mice

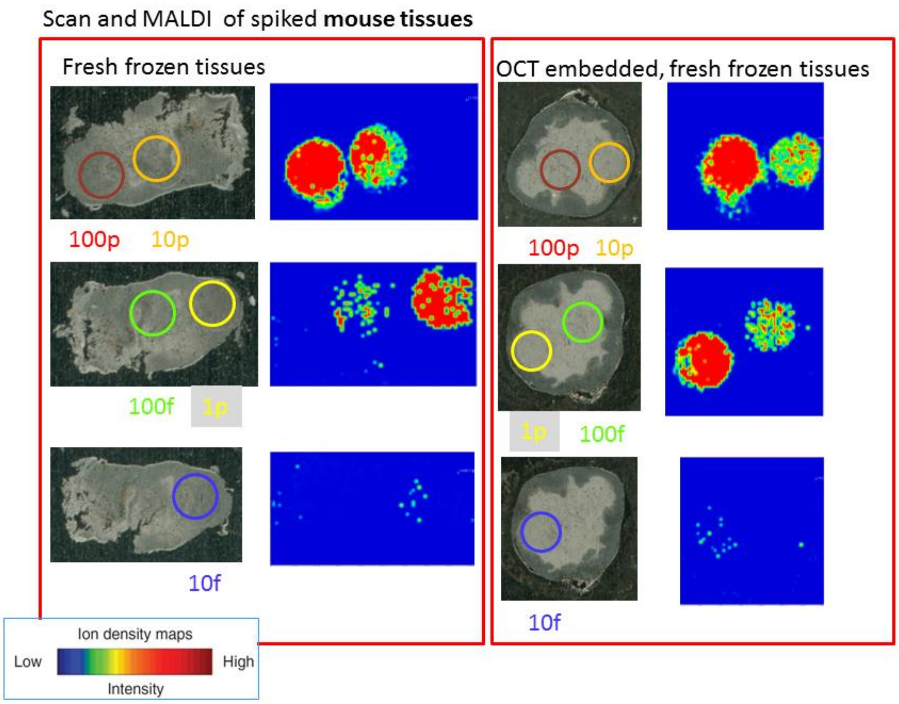

The optimized MALDI-MSI method developed to image veliparib was not sensitive enough to enable simultaneous acquisition of gadolinium within the same analytical run.

In this study, the LC-MS data were used for absolute quantification, whereas the MALDI-MSI data were used only to assess heterogeneity in spatial distribution within the tissue. Manual application of matrix was performed by the same trained user throughout all experiments to minimize variability. In addition, we used an internal standard added to the matrix to account for any differences in signal intensities per day of analysis. To avoid bias and person-to-person variability in the data-analyis, regions of interest were drawn on the optical tissue scan (defining necrotic and non-necrotic areas) without prior reference to the extracted veliparib MALDI-MS image.

To assess the correlation between veliparib pixel intensity by MALDI-MSI and concentrations quantified by LC-MS we the veliparib penetration was assessed by performing LCMS tissue extracts taken from an (approximately) 1mm tissue slice adjacent to the sections collected for MALDI-MSI and histology in treated HCC70 xenografts and select tumor 'rim' and 'center'. The observed mean MALDI-MSI pixel intensity was correlated with quantitative LC-MS results per dose level and in both tissue locations (Figure S3, $R^2 = 0.773$, P=0.013).

Figure S3: Correlation between tissue quantification in tumor using LC-MS and MALDI-MSI in which specifically tumors were sectioned of the same part of the tumor tissue and rim and center were quantified by LC-MS or visualized by MALDI-MSI

Specificity of the internal standard**:**

In order to assess whether iniparib was a suitable internal standard to address the Matrix effect in different parts of the tumor we compared it with normalization to the total ion chromatogram (TIC). TIC normalization has previously been used to minimize matrix and biological interference in MS image acquisition. The TIC mass range of 200-500 was selected which did not include the [M+H]+ [M+Na]+ and [M+K]+ DHB peaks.[3] We compared the pixel intensity of 4 images (example in Figure S4) and correlated the pixel intensity of veliparib in rim and center of the tumor, normalized to either iniparib internal standard or normalized using the TIC, Figure S5. The high correlation between TIC and internal standard normalized values adds further support that iniparib was a suitable internal standard for these experiments (Supplement Figure 4, R^2=0.90). MALDI-MSI data when normalized to iniparib, also correlated well with quantitative LCMS data from immediately-adjacent

tissue (Figure S5). The use of an internal standard for normalization only accounts for tissue-specific ionization effects and not for the extraction of the analyte drug from the tissue section. For this reason, future experiments will incorporate the use of deuterated veliparib as the internal standard and validation by comparison to adjacent microdissected tissue areas quantified by LCMS.

Figure S4) Example image of ID 965 raw image data normalized over total ion count (TIC) and over iniparib shows similar iniparib ion signal distribution throughout the tissue for both methods.

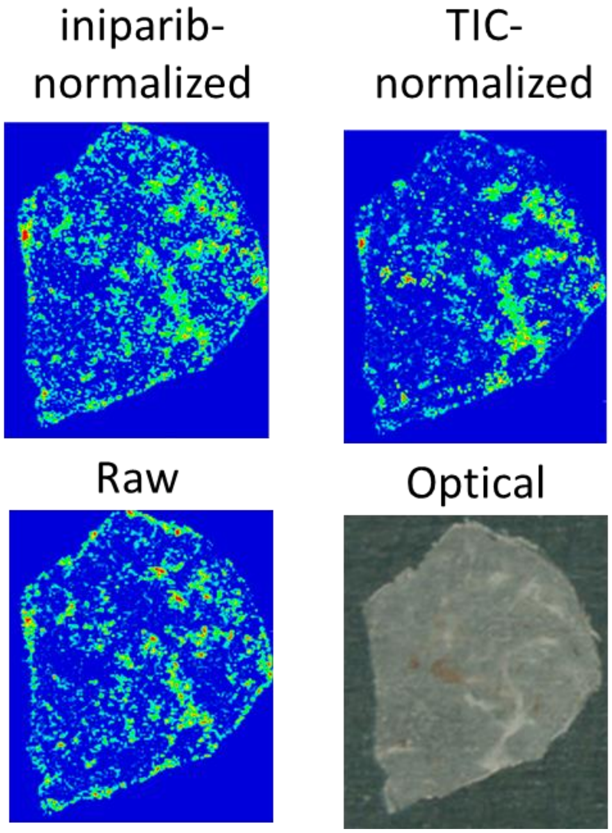

Figure S5) Correlation of the pixel intensity of veliparib in rim and center of the tumor, normalized to either internal standard or normalized using the total ion count (TIC)

Assess heterogeneity in drug penetration

Figure S6) Spatial distribution of veliparib low dose 20mg/kg **(a)** and high dose 60mg/kg **(b)** using 12 µm tissues from mice xenografted with three TNBC cell lines, three hours after the last dose. H&E (left) and MALDI-MSI (middle) of veliparib of each xenograft tumor are shown for two mice per cell type. The MALDI-MSI image of the muscle tissue of the same animal (right) is shown for comparison of variability in veliparib distribution between tissues. The MALDI-MSI images of both doses are presented on the same scale.

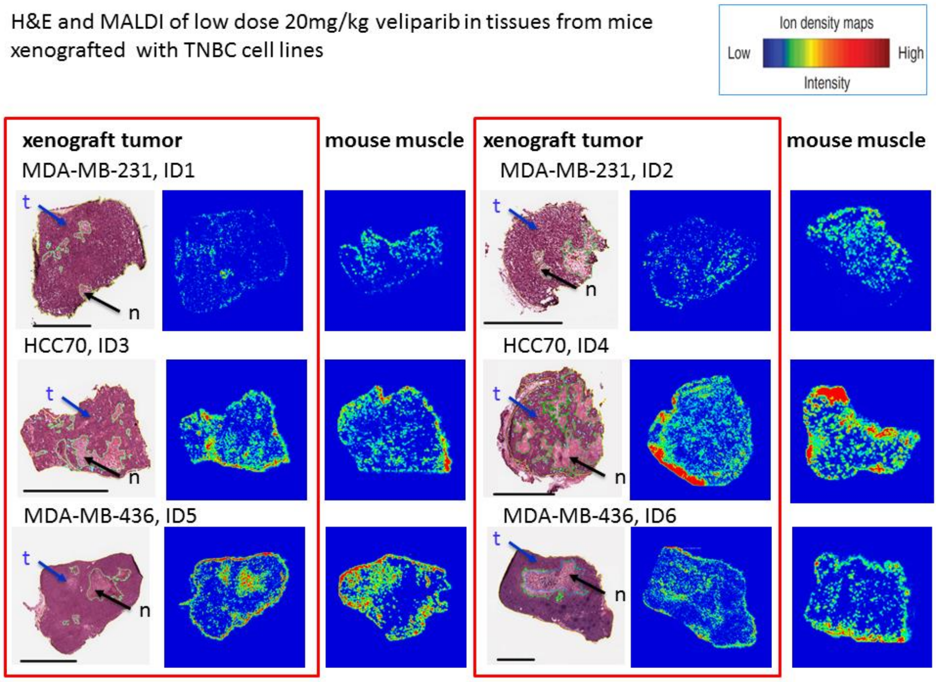

a)

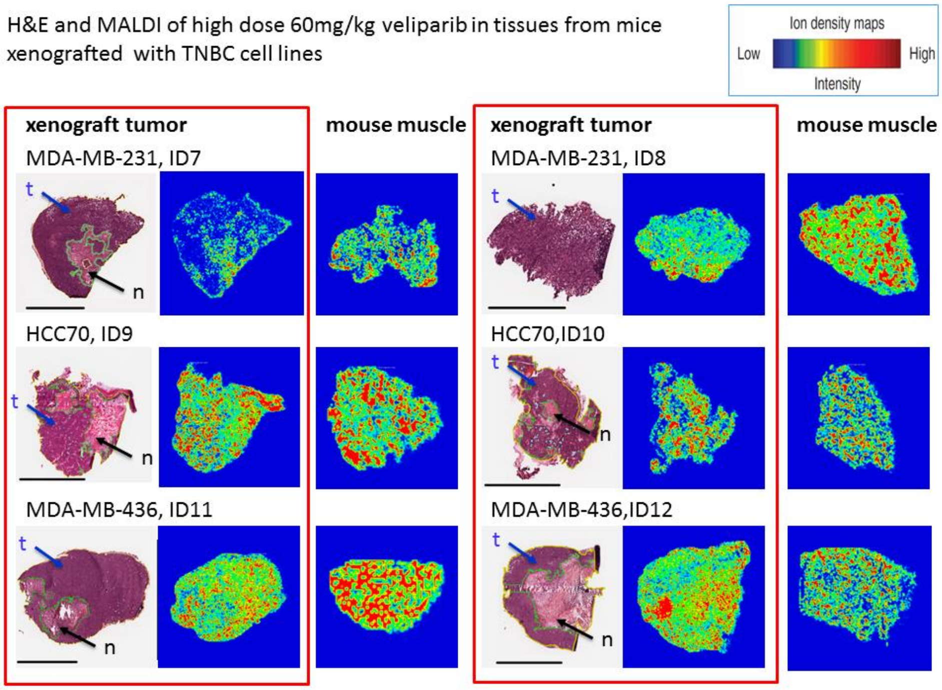

b)

Figure S7) Intra-individual variability after high dose 60mg/kg administration was assessed by studying drug uptake in two bilateral HCC70 xenografts, obtained1.5h* or 3 hours after the last dose **(a).** Two examples of accumulation of veliparib in adipose tissue in some samples (b)

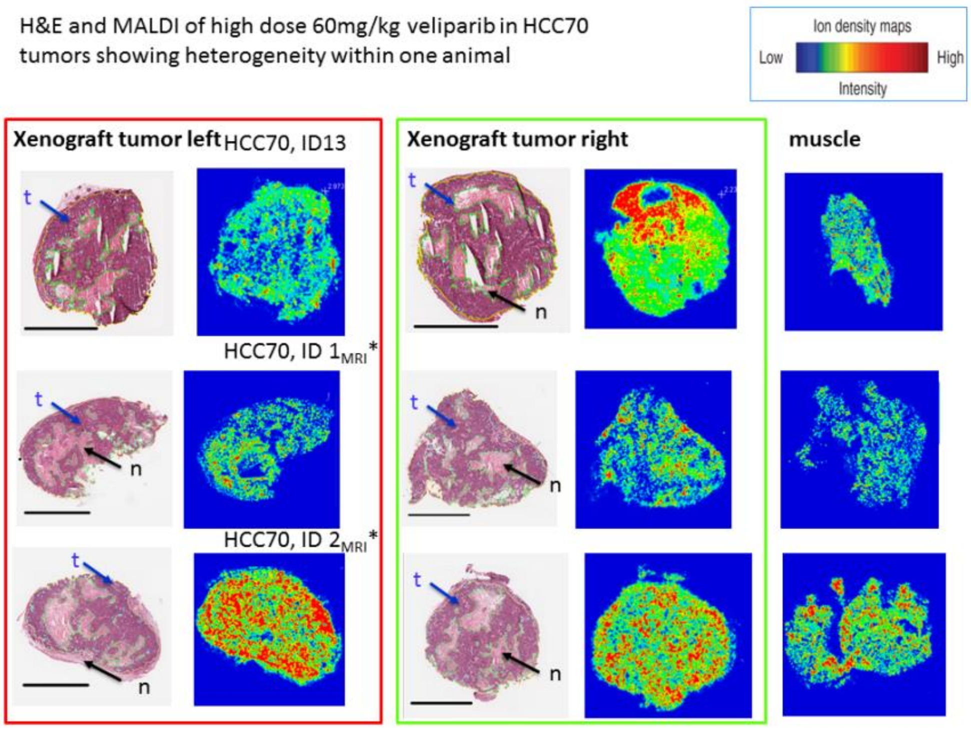

a)

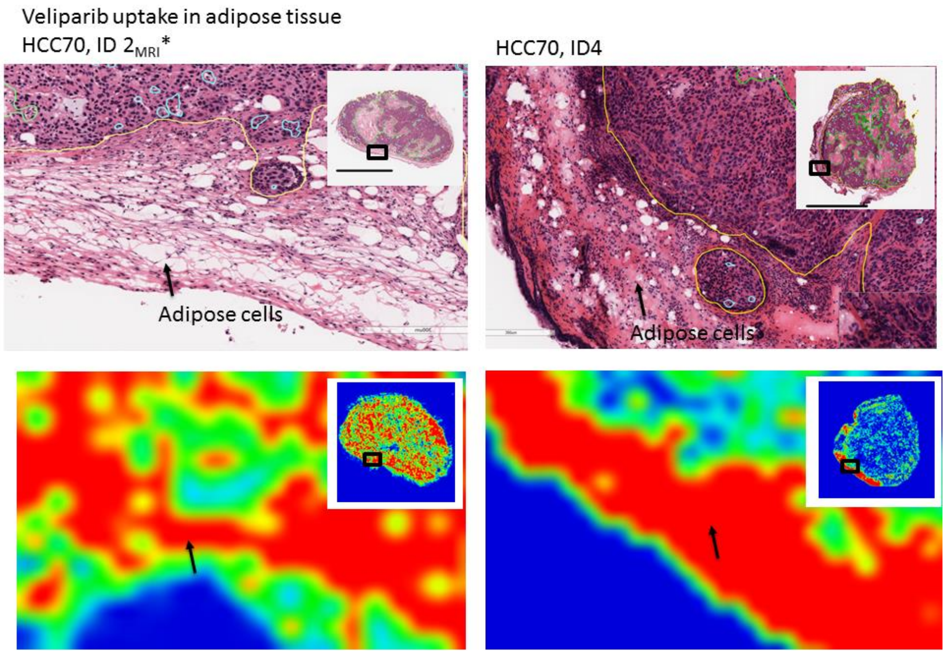

b)

b). Technical aspects of the ICP-MS method development.
PBMCs were extracted from whole blood using by centrifugation. Lysis of red blood cells was performed as needed, then PMBCs were washed and resuspended in

A limited number of 5-15 mL heparinised or EDTA whole blood samples from the PK study talazoparib/carboplatin phase 1 study were included. Following centrifugation for 15 min at 1,000 g, plasma was extracted and the PBS was added to a total volume of 9.5 ml to the remaing fraction. PMBCS were extracted using density-gradient centrifugation with Ficoll-Paque Plus® (Pharmacia, Uppsala, Sweden) at room temperature for 20 min at 400 *g*. PBMCs were washed twice with ice-cold PBS and centrifuged at 800 G for 5 minustes at 4 °C. Cells were counted and ther resuspended in 1 mL buffer containing 10 mmol L−1 Tris- HCl, 2.3% (w/v) NaCl, and 2 mmol L−1 EDTA disodium salt at pH 7.3.

<u>DNA extraction procedure:</u>

The DNA extraction was based on previous publications. [4] In short, 44 µL 1 mol L −1 NH4HCO3, 20 µL 20% (w/v) SDS, and 6 µL 1% (w/v) proteinase K solution were added successively, and incubated for 24hours using a thermomixer. After the digestion was complete, 146 µL saturated 6 mol L−1 NaCl was added to each tube and the tubes were shaken vigorously to precipitate proteins. The tubes were centrifuged for 10min at 2000 g and the supernatant containing the DNA was transferred to another tube. Subsequently, the supernatant was shaken, centrifuged, and transferred to another tube. Following this, 1.5ml absolute ethanol was added to precipitate the DNA and centrifuged from 30 minutes at 21 Kg at 4°C. The DNA was washed twice with 75% ethanol and was subsequently dissolved in 30 µL water. DNA concentrations were analyzed by measuring the absorbance at 260 nm using Nanodrop 1000 spectophotometer (NanoDrop).

<u>Carboplatin adduct quantification:</u>

The quantity of the carboplatin-adducts per mg DNA was quantified (Figure S8) using a previously validated ICP-MS method.[4]

<u>Figure S8: Example graph of mass spectrum of platinum in DNA extraction by ICP-MS.</u>

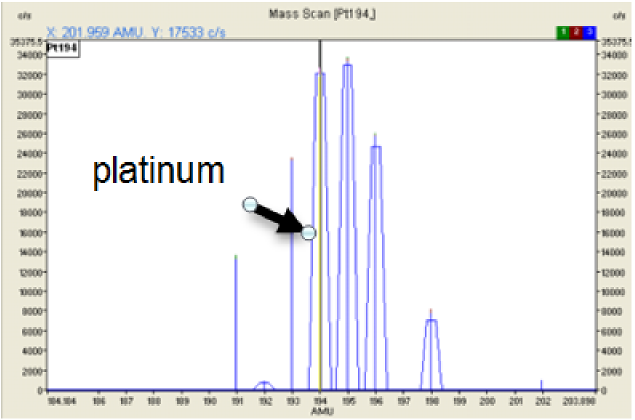

c) Technical aspects of the LC-MS method development.

Sample preparation:

Plasma samples (50 µl) were prepared using protein precipitation with acetonitrile. Small tumor samples (average 44 mg) were pulverized using Biopulverizer R (Biospec Bartlesville, OK, USA). Volume and weight of the pulverized tumor was determined. Veliparib was then extracted using cell lysis buffer (Cell Signaling Technology, Danvers, MA,USA), complemented with Halt Protease & Phosphatase (ThermoScientific, Atlanta, GA,USA). Protein precipitation of the tissue lysates was performed with acetonitrile. Oxaliplatin (Tzchem, Framingham, MA,USA) and iniparib (Sigma-Aldrich, Milwaukee, USA) were used as internal standards.

Veliparib/carboplatin quantification:

Carboplatin and veliparib were measured using LC-MS (Applied Biosystem Systems 3200 LC-MS/MS, Toronto, Canada), with electrospray ionization (ESI), using a Xterra® MS C18 3.5 µm column (Waters, Milford, MA, USA). The mobile phase contained 50:50 methanol and water with 0.1%, formic acid. Concentration of veliparib in tissues were calculated in mg/g and transferred to mg/L by estimating the average density of the livers, muscles and tumors.

Figure S9: Example graph of mass spectrum of veliparib and carboplatin and the internal standards in plasma by LC-MS.

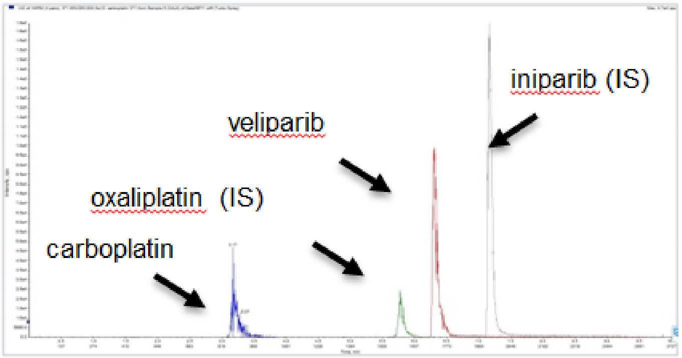

Method development/validation

The limit of quantification was 15 ng/ml for veliparib and 156 ng/ml for carboplatin. The calibration curves showed high accuracy (R^2= 0.9994 for veliparib, R^2=0.9984 for carboplatin).

Repetition of the tissue extraction and quantification from two tissue sections of the same animal showed consistent results R^2=0.874 as shown in Figure S10.

Figure S10) Repetition quantification from two tissue sections of the same animal to show consistence of results when repeating veliparib extraction (performed by LCMS).

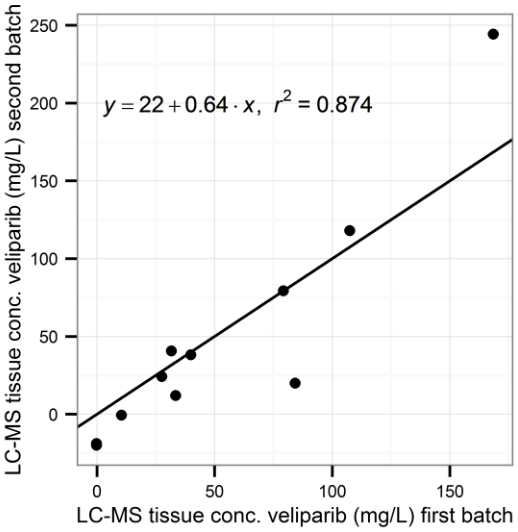

d) Dynamic contrast-enhanced MRI (DCE-MRI) experiments

Animal preparation:

Mice were anaesthetized with 2% isoflurane. For DCE-MRI, 0.43 mmol/kg of gadopentetate dimeglumine (Magnevist, Bayer Healthcare Pharmaceuticals, Berlin, Germany) contrast agent was administered intravenously through a tail vein injection.

Data-analysis:

Data were acquired on a 7 T horizontal bore MRI scanner (Horizontal Bore Varian MR System, Varian/Agilent, California) using a 38mm coil. T1-weighted gradient echo images were acquired for 60 minutes prior to, during and after contrast agent injection (TR= 15 ms, TE= 3.1 ms, flip angle=35° in plane spatial resolution=200 µm and slice thickness of 1 mm, 6 second temporal resolution).

DCE-MRI data visualization:

DCE-MRI processing and analysis was performed by plotting signal intensity versus time and maps of gadolinium distribution in the tumor, center and rim of the tumor; generated from DCE-MRI DICOM images using in-house IDL software as described previously.[5] The percent enhancement (PE) was calculated for each region of interest. The wash out was calculated using the observed signal enhancement between 15 and 25 minutes after gadolinium -infusion, using the formula: wash out (min^{-1})=-(log (S25minutes)-log (S15minutes))/(25-15)*2.303). The area under the gadolinium -signal enhancement time curve (AUC) was calculated using the start to the 25 minutes post infusion using the PK package in R, (version 1.3-2, R Thomas Jak (2015).

e) Supplements of the PK-model development.

Table S1: The parameter estimates of the pharmacokinetic model of veliparib pharmacokinetics in plasma and tumor

Structural model	Estimate	RSE	Shrinkage
**Plasma PK Veliparib**			
Vmax (mL/h)	1.86	46%	
V (mL)*	10.1	80%	
KA (h^{-1})	2.11	14%	
V2 (mL)	0.0168	44%	
Q tumor (ml/h)	0.0694	39%	
KM (mg/mL)	26.2	57%	
**Tumor PK Veliparib**			
Rate (KPT, ml/h)	900		
MDA.MB-231 uptake (ratio, RPT)	36%	7%	
HCC70 uptake (ratio, RPT)	75%	23%	
MDA.MB-436 uptake (ratio, RPT)	46%	40%	
**Random variability Veliparib**			
Interindividual variability on F plasma	13%	22%	23%
Interindividual variability on tumor penetration	37%	21%	24%
Intra-animal error tumor	28%	21%	19%
proportional residual error plasma	13%	42%	8%
proportional residual error tumor	23%	19%	7%
**Plasma PK Carboplatin**			
CL (mL/h)	34.2	9%	
Vd (mL)	17.5	12%	
t1/2 (h)	0.35		
Interindividual variability on CL	6%	30%	33%
proportional residual error plasma	41%	22%	9%

Plasma PK veliparib: $\frac{Dp}{Dt} = KA * A_{absorption} - \frac{VMAX*CP}{KM+CP} - \frac{Q}{Vcentral} * A_{central} + A_{peripheral} * \frac{Q}{Vperipheral}$

Tumor PK veliparib: $\frac{DT}{Dt} = KPT * (CP - CT) - KPT * CT$

Figure S11: *Plasma PK model derived from mice was used to predict plasma and tumor exposures in patients using veliparib 50 mg twice daily. In the grey boxes the data (either literature data or derived from the current study) are specified that were used for the model building and simulation. For the simulations in patients, the patient-based parameters are indicated in boldface (i.e.K, Ka, Vd), whereas those assumed to be equivalent to the preclinical values are in regular font. In this model, we assumed that veliparib penetration in mouse TNBCs was the same as that in patients. The population PK model published by Salem et al.*[6] *was used to predict plasma concentrations in patients. Inter-individual variability in tumor drug exposure among patients was considered similar to other hydrophilic cancer drugs such as epirubicin, assessed previously in 12 patients.* [7]

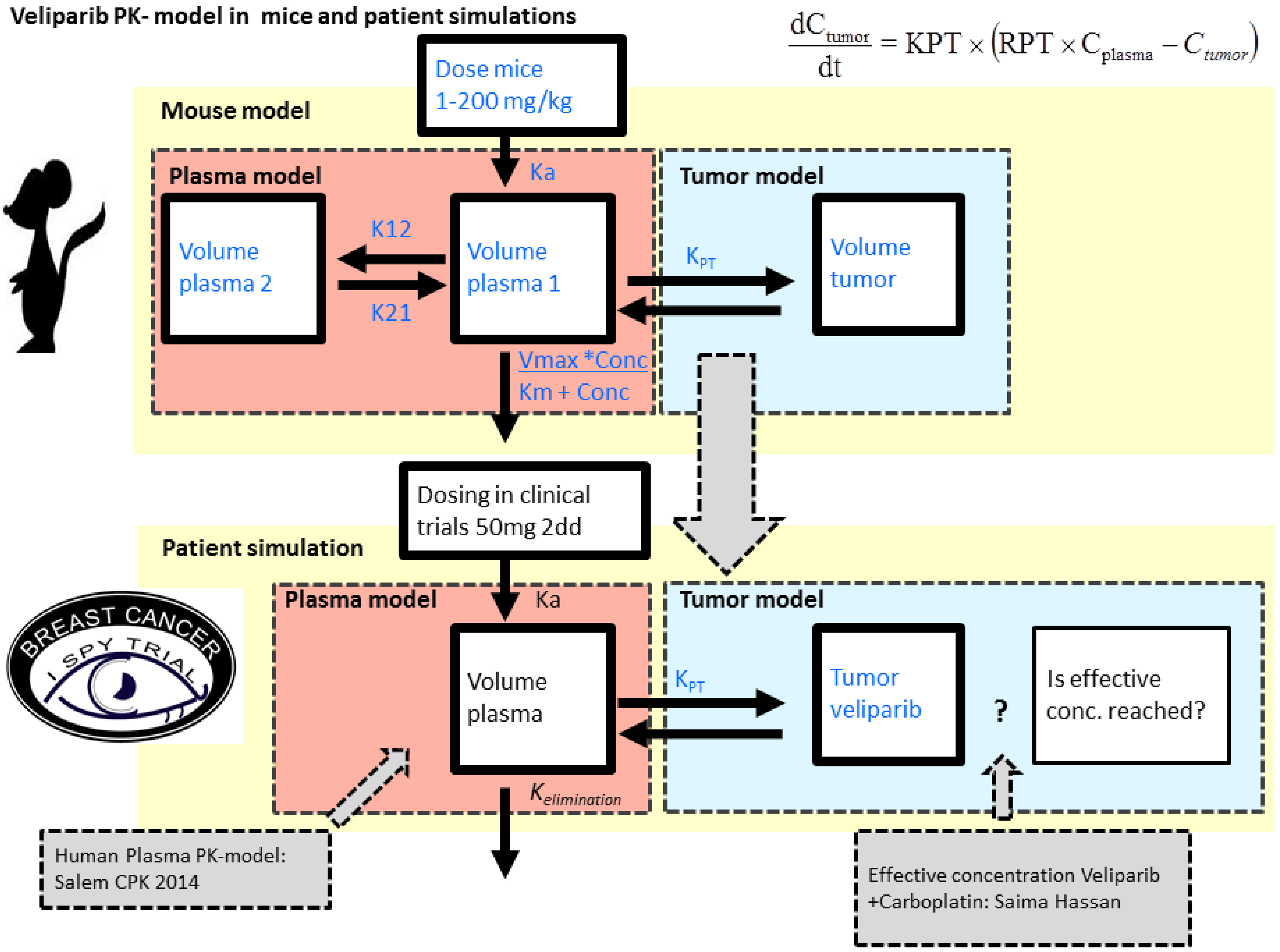

Supplementary Table 2: PK patients estimates carboplatin and platinum adducts

**Structural model PK**	**Estimate**	**RSE**
**Plasma PK Carboplatin**		
CLday1 (L/h)	5.2	10 %
CLday15 (L/h)	5.33	13 %
V1 (L)	0.935	49 %
Q (L/h)	0.324	61 %
V2 (L)	52.8	25 %
Q2 (L/h)	16.7	29 %
V3 (l0	7.97	9%
**Carboplatin adducts**		
RPT (%)		
gBRCA 1/2	0.40%	77 %
non-carriers	1.20%	56 %
KPT (L/h)	0.0156	2%
**Random variability PK**		
Inter-individual variability on Platinum adduct formation (RPT)	317%	
proportional residual error plasma carboplatin	28%	26 %
proportional residual error adduct formation	110%	6%

Platinum adduct formation: $\frac{dC_{adducts}}{dt} = KPT \times \left(RPT \times C_{plasma} - C_{adducts}\right)$

CL=clearance, V=volume of distribution, KA= rate of absorption, RPT= ratio of carboplatin adduct formation, KPT= rate of carboplatin adduct formation, gBRCA = germline BRCA carrier

Figure S12 Carboplatin concentration-time curves in plasma PK in 3 patients

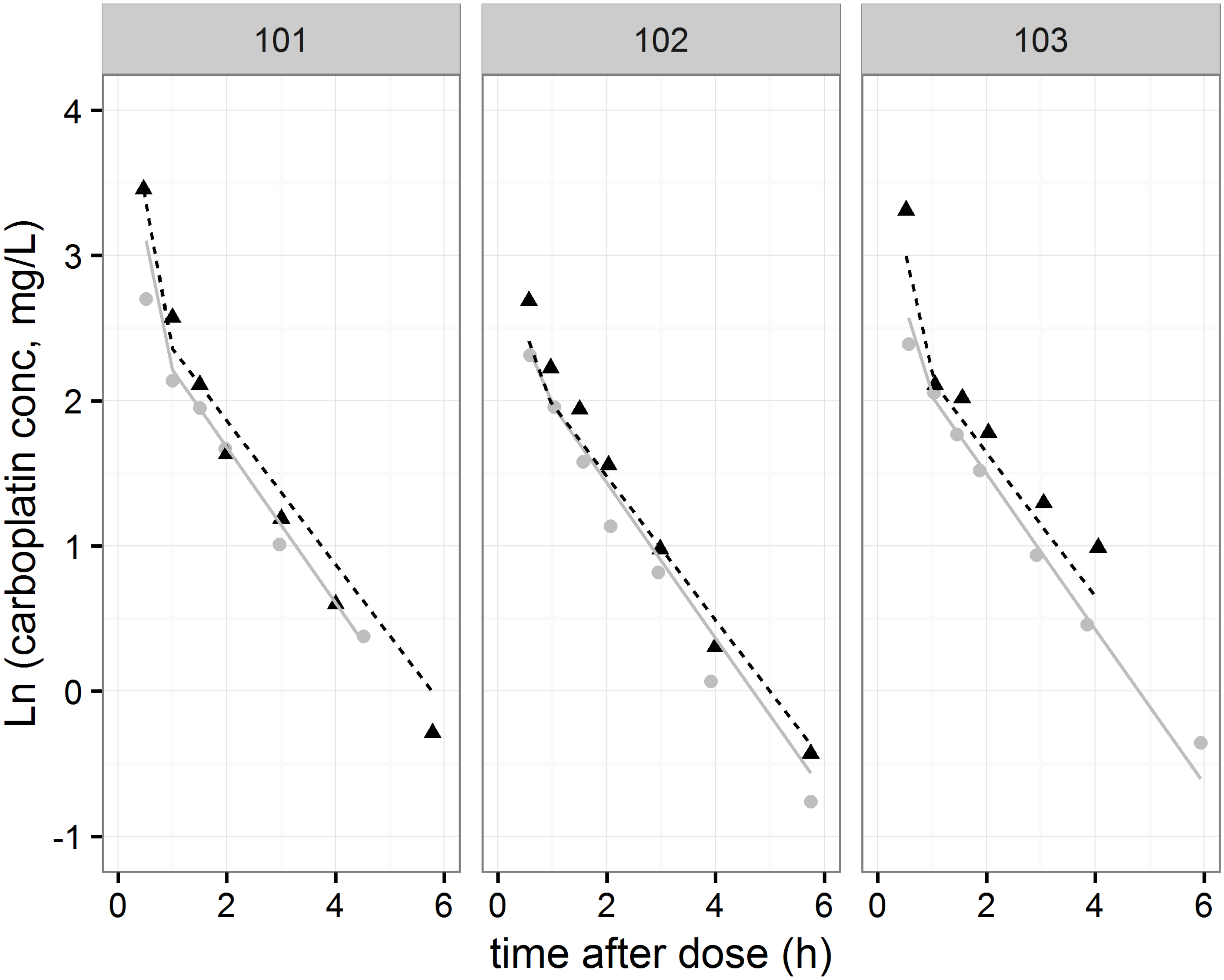

Figure S13 Platinum adducts at baseline at patients with prior platinum treatment versus no prior platinum treat ment: median 0.325 pg/L (range 0.007 – 2.74 pg/L) vs 4.935 pg/L (range 0.091-867), p-value = 0.007898.

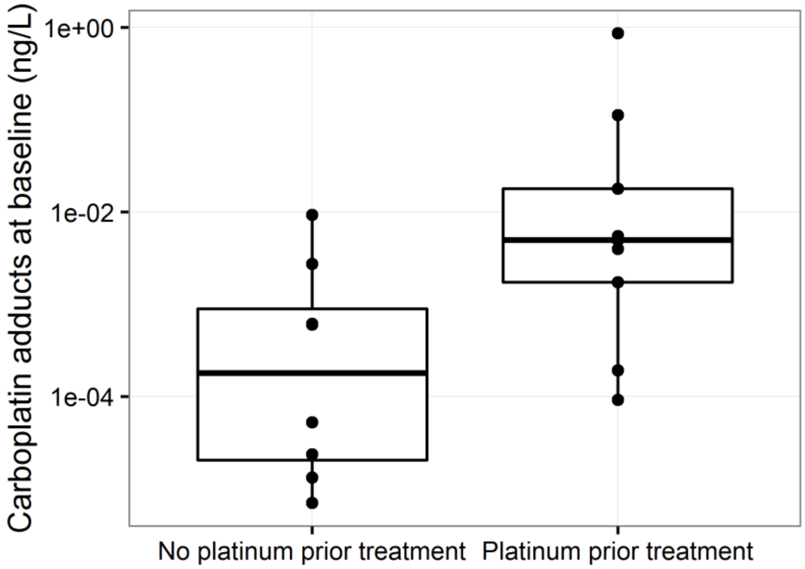

Figure S14**:** Carboplatin adducts observed in all patients: The patients with the highest adduct formation in PBMCs may potentially show more severe toxicity (although in this small dataset this could not be tested). Observed and model-predicted carboplatin adduct concentration-time profiles in 5 gBRCA patients and 9 non-carriers and 5 patients with unknown carrier status. These plots suggest that lower carboplatin adduct formation was observed in in BRCA 1/ 2 mutated patients (dark red) compared to non-carriers (green) or unknown patients (blue). **Figure Notes:** The observed data (dots), median predicted values (blood counts or adducts) in BRCA 1/ 2 mutated patients (dark red) , non-carriers (green) and patients in which germline mutations were unknown (blue). The grey arrow shows the timepoint where dosing of the PARPi talazoparib was initiated.

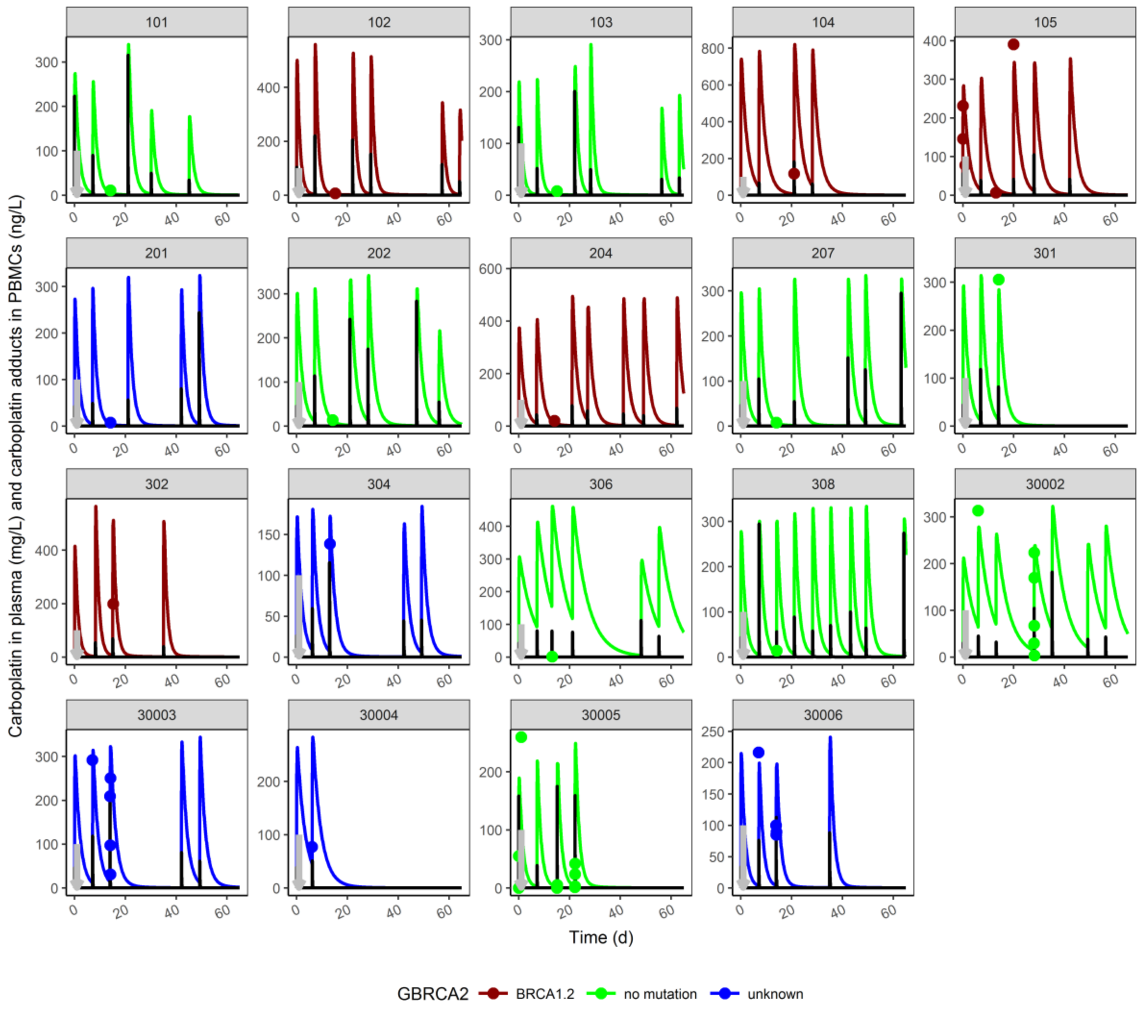

1 Reiner T, Lacy J, Keliher EJ, *et al.* Imaging therapeutic PARP inhibition in vivo through bioorthogonally developed companion imaging agents. *Neoplasia (New York, NY)* 2012; **14**: 169–77.

2 Solomon B, Binns D, Roselt P, *et al.* Modulation of intratumoral hypoxia by the epidermal growth factor receptor inhibitor gefitinib detected using small animal PET imaging. *Molecular cancer therapeutics* 2005; **4**: 1417–22.

3 Fonville JM, Carter C, Cloarec O, *et al.* Robust data processing and normalization strategy for MALDI mass spectrometric imaging. *Analytical chemistry* 2012; **84**: 1310–9.

4 Brouwers EEM, Tibben MM, Pluim D, *et al.* Inductively coupled plasma mass spectrometric analysis of the total amount of platinum in DNA extracts from peripheral blood mononuclear cells and tissue from patients treated with cisplatin. *Analytical and bioanalytical chemistry* 2008; **391**: 577–85.

5 Wilmes LJ, Pallavicini MG, Fleming LM, *et al.* AG-013736, a novel inhibitor of VEGF receptor tyrosine kinases, inhibits breast cancer growth and decreases vascular permeability as detected by dynamic contrast-enhanced magnetic resonance imaging. *Magn ResonImaging* 2007; **25**: 319–27.

6 Salem AH, Giranda VL, Mostafa NM. Population pharmacokinetic modeling of veliparib (ABT-888) in patients with non-hematologic malignancies. *Clinical pharmacokinetics* 2014; **53**: 479–88.

7 Hunz M, Jetter A, Warm M, *et al.* Plasma and tissue pharmacokinetics of epirubicin and Paclitaxel in patients receiving neoadjuvant chemotherapy for locally advanced primary breast cancer. *Clinical pharmacology and therapeutics* 2007; **81**: 659–68.
